# Intracellular trafficking of HLA-E and its regulation

**DOI:** 10.1084/jem.20221941

**Published:** 2023-05-04

**Authors:** Wanlin He, Ester Gea-Mallorquí, Huw Colin-York, Marco Fritzsche, Geraldine M. Gillespie, Simon Brackenridge, Persephone Borrow, Andrew J. McMichael

**Affiliations:** 1 Nuffield Department of Medicine, Center for Immuno-Oncology, University of Oxford, Oxford, UK; 2 Kennedy Institute of Rheumatology, University of Oxford, Oxford, UK

## Abstract

Interest in MHC-E–restricted CD8^+^ T cell responses has been aroused by the discovery of their efficacy in controlling simian immunodeficiency virus (SIV) infection in a vaccine model. The development of vaccines and immunotherapies utilizing human MHC-E (HLA-E)–restricted CD8^+^ T cell response requires an understanding of the pathway(s) of HLA-E transport and antigen presentation, which have not been clearly defined previously. We show here that, unlike classical HLA class I, which rapidly exits the endoplasmic reticulum (ER) after synthesis, HLA-E is largely retained because of a limited supply of high-affinity peptides, with further fine-tuning by its cytoplasmic tail. Once at the cell surface, HLA-E is unstable and is rapidly internalized. The cytoplasmic tail plays a crucial role in facilitating HLA-E internalization, which results in its enrichment in late and recycling endosomes. Our data reveal distinctive transport patterns and delicate regulatory mechanisms of HLA-E, which help to explain its unusual immunological functions.

## Teaser

Understanding the unique features of immune functions of HLA-E to inform future clinical exploitation requires detailed knowledge of HLA-E transport. He et al. characterize the basic trafficking patterns of HLA-E within cells and how this trafficking is regulated.

## Introduction

MHC-E is a highly conserved non-classical major histocompatibility complex class I (MHC-I) molecule. Although HLA-E (human MHC-E) cell surface expression is universal, it is normally low compared with classical HLA-Ia molecules (D’Souza et al. 2019). The main function of HLA-E is to present a highly conserved peptide VL9 (VMAPRTL/VV/L/FL), which is derived from the leader sequences of other HLA-I (HLA-A/B/C and G) molecules, to CD94/NKG2 receptors on natural killer (NK) cells (Borrego et al., 1998; Braud et al., 1998a). Thus, MHC-E regulates NK cell functions, a role that is conserved across mammals.

MHC-E can also present different pathogen-derived peptides to CD8^+^ T cells, though reports are scarce compared with classical MHC-Ia molecules. HLA-E can activate CD8^+^ T cells through the presentation of *Mycobacterium tuberculosis* (Mtb) peptides (Heinzel et al., 2002). Among the 36 Mtb-derived peptides shown to bind to HLA-E, 11 are recognized by CD8^+^ T cells (Joosten et al., 2010). The CD8^+^ T cells activated by these peptides in Mtb infection exhibit HLA-E–restricted cytotoxic activity against mycobacterium-infected cells (van Meijgaarden et al., 2015). HLA-E can also present *Salmonella typhi* (*S. typhi*) peptides to CD8^+^ T cells and inhibit *S. typhi* pathogenesis (Fresnay et al., 2016; Rudolph et al., 2019; Salerno-Gonçalves et al., 2004). A BZLF1 peptide derived from the EBV can also bind to HLA-E and activate CD8^+^ T cells (García et al., 2002; Ulbrecht et al., 1998). HLA-E–restricted presentation of human cytomegalovirus (HCMV) UL40-derived peptide, with an identical sequence to the human VL9 peptide, can stimulate specific CD8^+^ T cells that effectively recognize and kill HCMV-infected cells (Mazzarino et al., 2005; Pietra et al., 2003). Recent studies of a simian immunodeficiency virus (SIV) vaccine that enabled 55% of rhesus macaques (RMs) to clear infection following an SIV challenge have sparked renewed interest in MHC-E (Hansen et al., 2011; Hansen et al., 2013; Hansen et al., 2016). This vaccine, vectored by a particular strain of rhesus cytomegalovirus (RhCMV68-1) with key gene deletions and engineered to express SIV genes, stimulates an MHC-E–restricted CD8^+^ T cell response that is critical for the effective and long-lasting protection observed in vaccinated RMs (Hansen et al., 2016; Malouli et al., 2021).

HLA-E–bound pathogen-derived epitopes offer potential advantages for vaccine-elicited CD8^+^ T cell responses. Many pathogens downregulate surface HLA-Ia molecules to evade immune recognition (Antoniou and Powis, 2008), which poses a significant challenge for vaccines targeting CD8^+^ T cell responses. However, HLA-E is usually resistant to such downregulation and is frequently upregulated, thus preserving the HLA-E–NKG2A axis, which enables pathogens to avoid NK cell–mediated lysis (McMichael and Picker, 2017). This retained or augmented surface expression ensures the efficacy of HLA-E–restricted CD8^+^ T cells if they can be elicited by vaccination. The typical low surface expression of HLA-E may prevent or limit natural HLA-E–restricted T cell priming in most infections, making prior immune escape unlikely and facilitating the therapeutic application of HLA-E–restricted T cells. There are only two dominant alleles of HLA-E that differ by a single amino acid substitution at position 107, which does not affect the peptide binding groove or the peptide repertoire (O’Callaghan et al., 1998). This lack of polymorphism means that HLA-E–restricted CD8^+^ T cell responses are shared between individuals, simplifying vaccine and immunotherapeutic design.

Understanding HLA-E transport pathways is essential for gaining a better understanding of the immunological roles of HLA-E and for exploring the HLA-E–restricted CD8^+^T cell responses for therapeutic purposes. Although relatively understudied, several lines of evidence suggest that HLA-E transport differs from that of HLA-Ia molecules. For some of the pathogens that elicit MHC-E–restricted T cell responses, MHC-E has been found to source pathogen-derived peptides in an unconventional manner. In infected macrophages, HLA-E–restricted presentation of Mtb peptides involves recycling HLA-E molecules instead of newly synthesized ones (Grotzke et al., 2009). Peptide loading occurs in Mtb phagosomes where proteins necessary for peptide loading are found and HLA-E molecules are enriched (Grotzke et al., 2009). In RhCMV68-1 vaccination, the processing of the endogenous VL9 peptide is blocked. However, an identical peptide derived from the Rh67 signal sequence associates with MHC-E in the ER without the aid of the transporter associated with antigen processing (TAP; Hansen et al., 2016; Verweij et al., 2021) and facilitates MHC-E antegrade transport. This process, which can occur despite CMV downregulation of MHC-Ia molecules and TAP (Hewitt et al., 2001; Lee et al., 2005; Park et al., 2004; Wiertz et al., 1996a; Wiertz et al., 1996b), is essential for the priming of the MHC-E–restricted, SIV-specific CD8^+^ T cells by the RhCMV68-1–vectored SIV vaccine (Verweij et al., 2021).

Other MHC-I–like molecules have been reported to undergo atypical antigen processing and transport pathways (Ogg et al., 2019). Surface CD1a-d molecules can be internalized and transported to distinct locations within the endosomal network for lipid loading (Gumperz, 2006). The endosomal trafficking pathways are determined by their cytoplasmic tail sequences, which vary amongst CD1 isoforms (Gumperz, 2006). Similarly, MR1 can load ligands coming from endocytosis, phagocytosis, or autophagy in endosomes, and then be recycled back to the cell surface (Lamichhane and Ussher, 2017). CD74 and HLA-DM, the chaperones important for HLA-II transport, might influence this unconventional trafficking of MR1 (Huang et al., 2008).

Here, we sought to characterize HLA-E trafficking patterns and the underlying regulatory mechanisms. The limited supply of high-affinity peptides, which resulted in ER retention and low surface stability of HLA-E, was found to be the primary cause of its low surface expression. The HLA-E cytoplasmic tail also contributed to its ER retention but had a more profound role in facilitating the rapid internalization of surface HLA-E molecules and their enrichment in late endosomes and recycling endosomes. These findings not only deepen our understanding of how HLA-E achieves its immunological functions through specialized transport regulation but also inform future rational strategies for the development of immunotherapies and vaccines that could exploit unconventional HLA-E–restricted CD8^+^ T cell responses.

## Results

### HLA-E is enriched in the ER, late endosomes, and recycling endosomes

**Figure 1.**
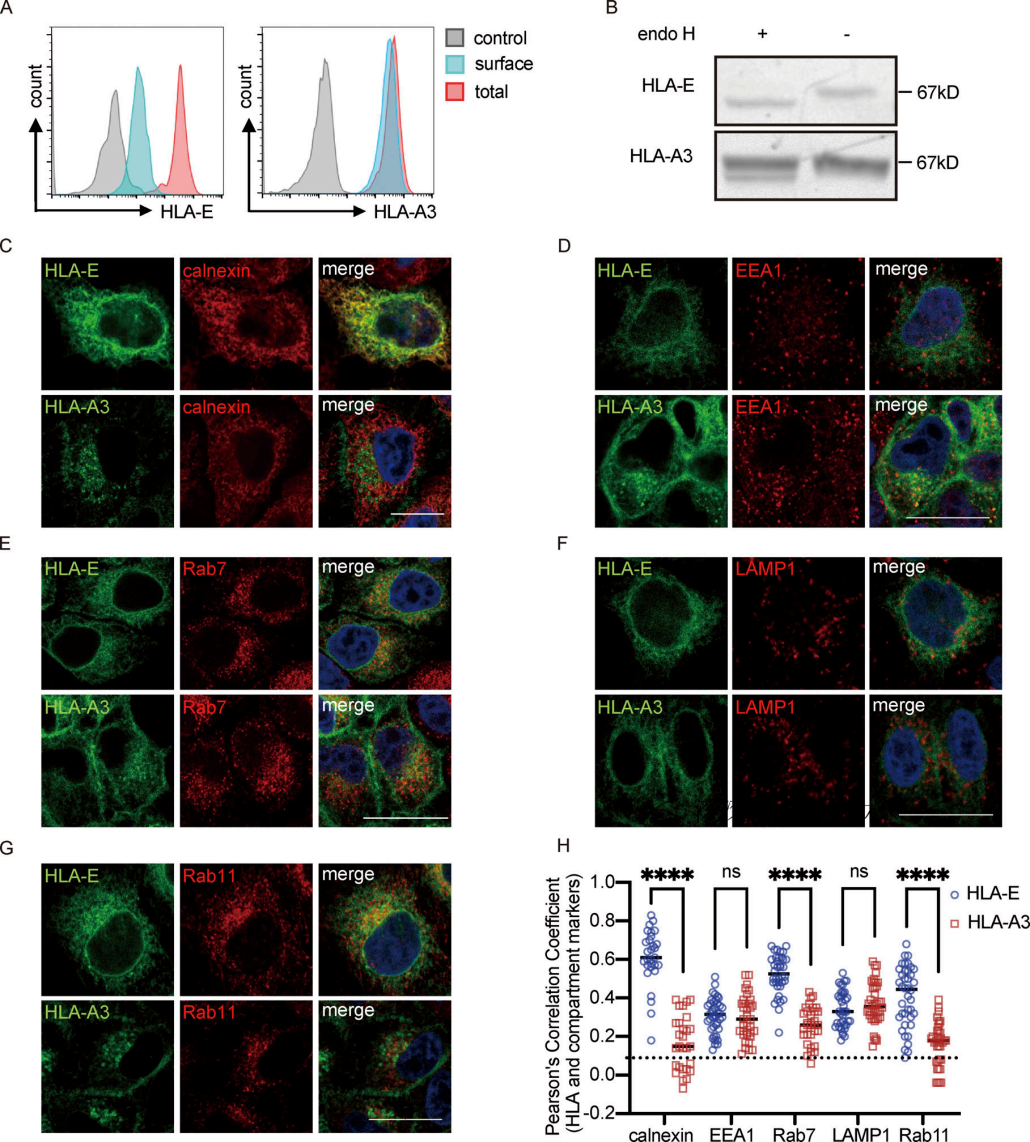
**Intracellular distribution of HLA-E. (A)** HeLa.E or HeLa.A3 were collected for flow cytometry analysis. Representative graphs of total expression (red) and surface expression (blue) of HLA-E or HLA-A3 are shown. MFI of the unstained sample (gray) was used as the negative control. MFIs shown here are representative of observations made in six independent experiments. **(B)** Lysates of HeLa.E or HeLa.A3 were treated with Endo H, followed by detection with immunoblotting using an anti-EGFP antibody. Figures shown here are representative of three independent experiments. **(C–G)** Representative micrographs of HeLa.E or HeLa.A3. After fixation and permeabilization, cells were stained with antibodies against marker proteins for ER (calnexin; C), early endosome (EEA1; D), late endosome (Rab7; E), lysosome (LAMP1; F), and recycling endosome (Rab11; G), followed by detection with Alexa568-conjugated secondary antibody. Scale bars = 20 μm. Micrographs shown here are representative of two independent experiments. **(H)** Quantification of colocalization of HLA-E or HLA-A3 with different marker proteins from Fig. 1, C–G. The PCC values of each cell and the mean values are shown with 20–40 cells per sample. Statistical analysis was performed using unpaired two-tailed Student’s *t* test with Welch’s correction. Asterisks show the statistical significance between indicated groups: ns, not significant; ****, P < 0.0001. Source data are available for this figure: SourceData F1.

**Figure S1.**
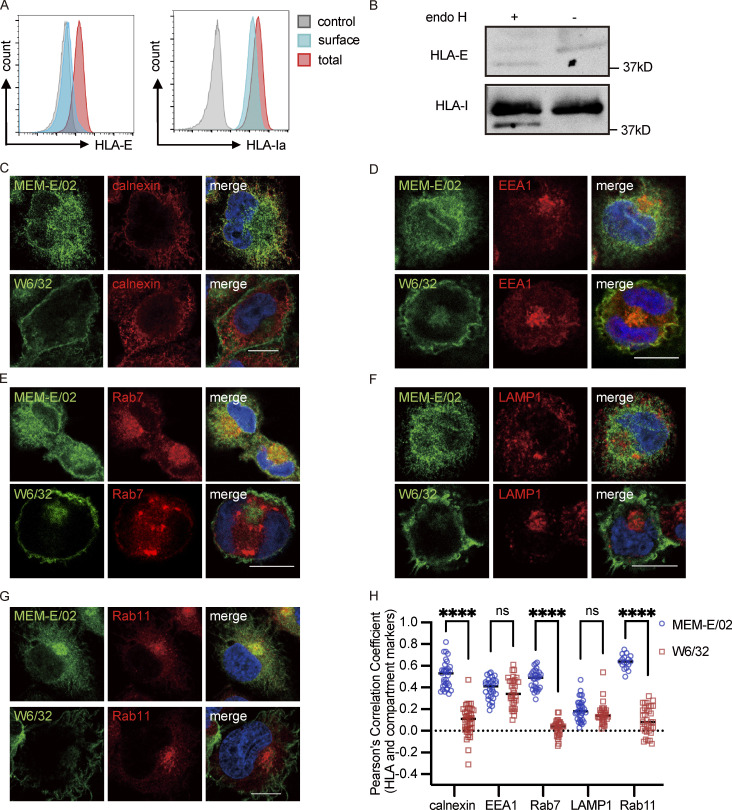
**Intracellular distribution of HLA-E in THP1-derived macrophages. (A)** THP1-derived macrophages were collected for flow cytometry analysis. Representative graphs of total expression (red) and surface expression (blue) of HLA-E or HLA-Ia are shown. MFI of the unstained sample (gray) was used as the negative control. MFIs shown here are representative of observations made in six independent experiments. **(B)** Lysates of THP1-derived macrophages were treated with Endo H, followed by detection with immunoblotting. Figures shown here are representative of three independent experiments. **(C–G)** Representative micrographs of THP1-derived macrophages. Cells were fixed, permeabilized, and co-stained with rabbit antibodies against different marker proteins (ER [calnexin; C], early endosome [EEA1; D], late endosome [Rab7; E], lysosome [LAMP1; F], recycling endosome [Rab11; G]), and mouse antibodies against different HLA-Imolecules (HLA-E [MEM-E/02], HLA-Ia [W6/32]). Cells were then stained with anti-rabbit Alexa568 and anti-mouse Alexa488 secondary antibodies. Scale bars = 10 μm. Micrographs shown here are representative of two independent experiments. **(H)** Quantification of colocalization of HLA-E or HLA-Ia with different marker proteins from Fig. S1, C–G. The PCC values of each cell and the mean values are shown with 30–40 cells per sample. Statistical analysis was performed using unpaired two-tailed Student’s *t* test with Welch’s correction. Asterisks show the statistical significance between indicated groups: ns, not significant; ****, P < 0.0001. Source data are available for this figure: SourceData FS1.

To characterize HLA-E intracellular localization and transport, we constructed HeLa cell lines stably expressing HLA-E*01:03 (HeLa.E) or HLA-A*03:01 (HeLa.A3) tagged with EGFP. HLA-A*03:01, which is not known to share the binding of peptides with HLA-E, was selected as the control. To examine the proportion of HLA-E expressed on the cell surface, we compared the mean fluorescence intensity (MFI) of permeabilized samples, which indicated the total protein expression level, to the MFI of surface staining. While HLA-A3 was mainly found on the plasma membrane in HeLa.A3 cells, HLA-E was mostly found intracellularly in HeLa.E cells (Fig. 1 A), which is consistent with previous studies (Coupel et al., 2007; Lee et al., 1998). Most HLA-E was sensitive to endoglycosidase H (Endo H) cleavage (Fig. 1 B), showing that the majority of HLA-E molecules were immature and stayed in the early secretory pathway, as previously described (Camilli et al., 2016). In contrast, most HLA-A3 was insensitive to Endo H cleavage, which corresponds to its intracellular distribution being strongly biased toward cell surface expression. The ER accumulation of HLA-E was confirmed by immunofluorescence imaging, as shown by the high Pearson correlation coefficient (PCC), indicating good colocalization of HLA-E with calnexin (Fig. 1, C and H). Similar results were obtained for endogenous HLA-E in PMA-differentiated THP1 cells that resemble macrophages (Fig. S1, A–C and H). We also investigated the distribution of HLA-E in different endosomal compartments. Both HLA-E and HLA-A3 were found in early endosomes (Fig. 1 D), late endosomes (Fig. 1 E), lysosomes (Fig. 1 F), and recycling endosomes (Fig. 1 G). While there was no apparent difference between HLA-E and HLA-A3 in early endosomes and lysosomes (Fig. 1, D, F, and H), HLA-E was more enriched in late endosomes and recycling endosomes, as evidenced by better colocalization with Rab7 and Rab11 compared with HLA-A3 (Fig. 1, E, G, and H). Endogenous HLA-E distribution in THP1-derived macrophages followed a similar pattern (Fig. S1, D–H).

Thus, HLA-E differs from HLA-A3 by enrichment in the ER as well as in late and recycling endosomes, with only a tiny fraction expressed on the cell surface.

### Antegrade transport of HLA-E and HLA-A3 is similar in HeLa cells

**Figure 2.**
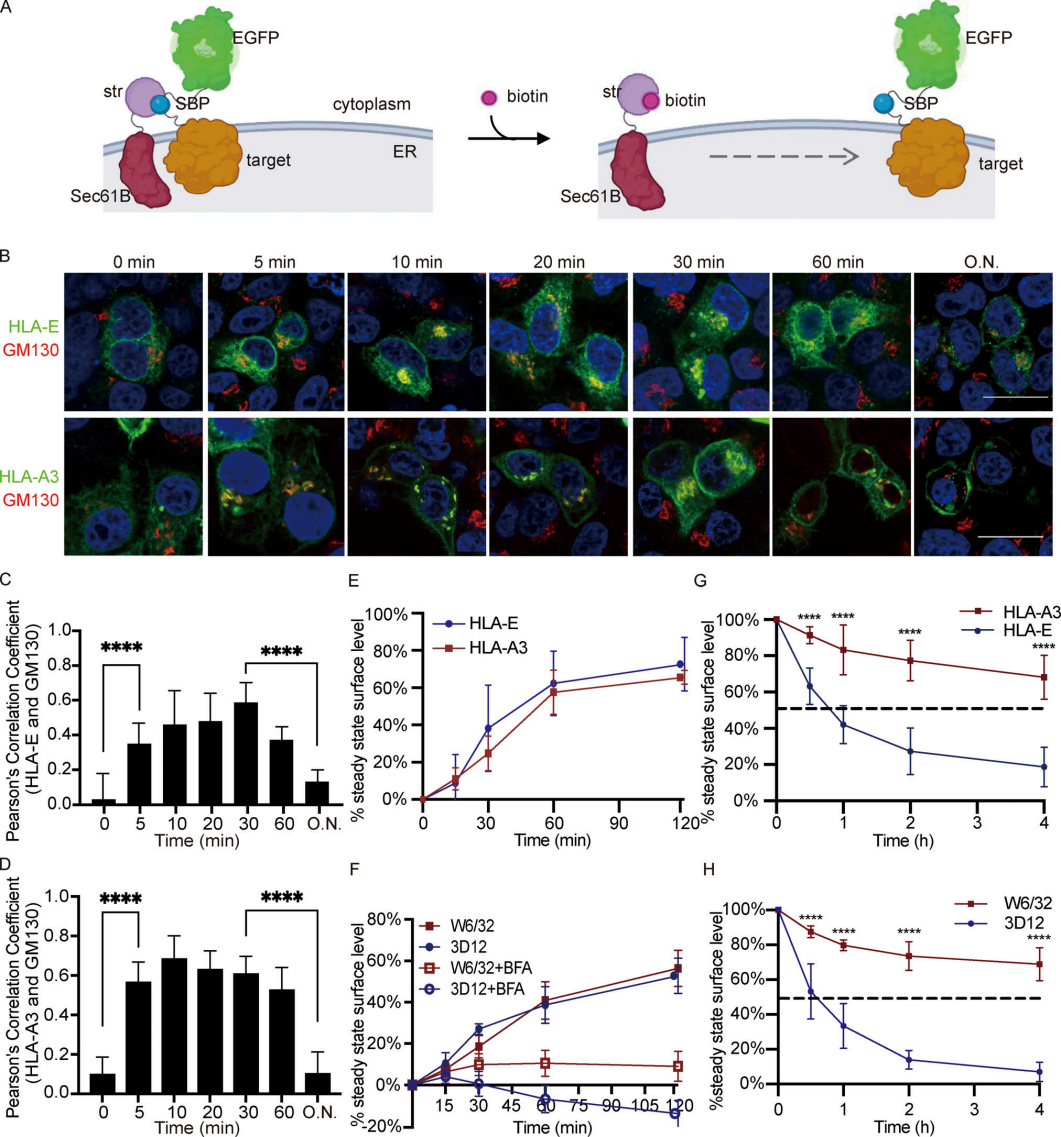
**Intracellular transport of HLA-E. (A)** Schematic diagram of the RUSH system. The reporter complex contains the target protein (HLA-E or HLA-A3) with SBP and EGFP fused to the C terminus. The hook contains streptavidin (str) fused to the N terminus of the ER-resident protein Sec61B, which retains the reporter complex in the ER through the interaction between SBP and streptavidin. Biotin binds to streptavidin and enables the trafficking of the target protein out of ER. The figure was adapted from Boncompain et al. (2012) and created with BioRender.com. **(B–D)** HeLa cells were transiently cotransfected with Sec61B-streptavidin and HLA-E_SBP_EGFP or HLA-A3_SBP_EGFP. At different time points after biotin addition, cells were fixed, permeabilized, and stained with an antibody against the Golgi marker protein GM130, followed by detection with an Alexa568-conjugated secondary antibody. **(B)** Representative micrographs of two independent experiments. Scale bars = 20 μm. **(C and D)** Quantification of Golgi colocalization for HLA-E (C) or HLA-A3 (D). O.N., overnight. At each time point, PCC was calculated for 10–20 individual cells, and the data are shown as mean ± SD (error bars). **(E)** HEK 293T cells were transiently cotransfected with Sec61B-streptavidin and HLA-E_SBP_EGFP (blue circle) or HLA-A3_SBP_EGFP (red square). At different time points after biotin addition, cells were collected for flow cytometry analysis. The stable cell surface MFI after the biotin addition overnight was set to 100%, the MFI before biotin addition was set to 0%, and the other values were normalized accordingly. Data were collected for three biological runs and are shown as mean ± SD (error bars). **(F)** Surface HLA molecules on THP1-derived macrophages were stripped off by citric acid, and then cells were incubated in media with (hollow) or without (solid) BFA for different time points before flow cytometry analysis. The average cell surface MFI for time point zero was set to 0%, the average cell surface MFI without acid stripping was set to 100%, and the following values were normalized as its percentage. Data were collected for three biological runs and are shown as mean ± SD (error bars). **(G and H)** HeLa cells stably expressing HLA-A3 (red square) or HLA-E (blue circle; F) or THP1-derived macrophages (G) were incubated in media containing BFA for different time points, and surface expression of HLA molecules was assessed. The average cell surface MFI for time point zero was set to 100%, and the following values were normalized as their percentage. The dashed lines represent 50%. Data were collected for three biological runs and are shown as mean ± SD (error bars). Statistical analysis was performed using unpaired two-tailed Student’s *t* test with Welch’s correction. Asterisks show the statistical significance between indicated groups: ****, P < 0.0001.

**Figure S2.**
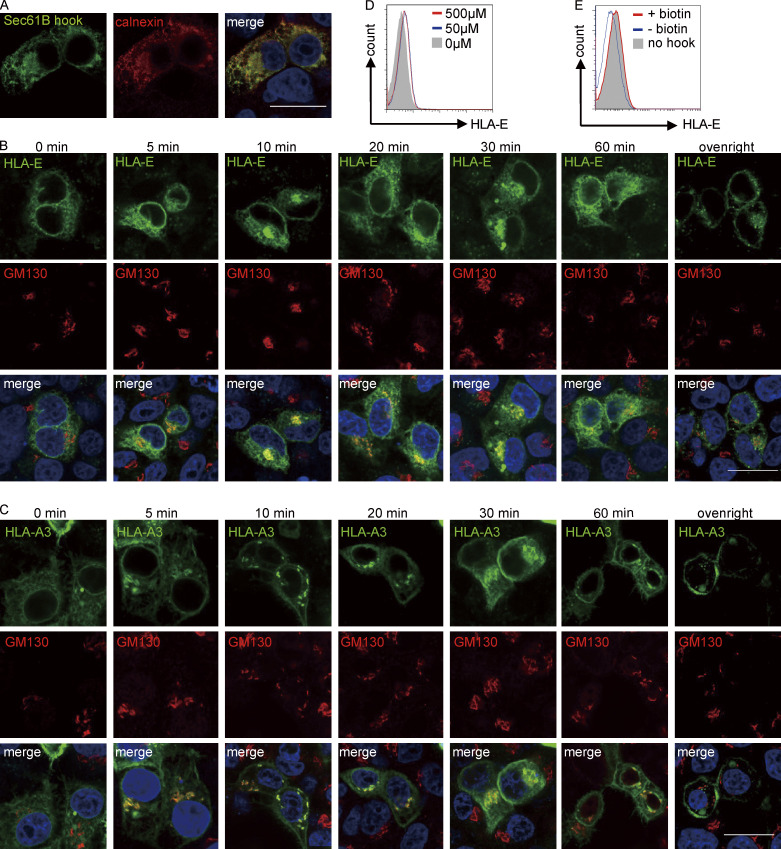
**Application of the RUSH system to study intracellular transport. (A)** Representative micrographs of HeLa cells transiently expressing the hook Sec61B_streptavidin. Cells were fixed, permeabilized, and stained with a mouse antibody against streptavidin and a rabbit antibody against the ER marker protein calnexin, followed by detection with anti-mouse Alexa488 and anti-rabbit Alexa568 secondary antibodies. Scale bar = 20 μm. Micrographs shown here are representative of two independent experiments. **(B and C)** Representative micrographs of HeLa cells transiently expressing Sec61B_streptavidin and HLA-E_SBP_EGFP (B) or HLA-A3_SBP_EGFP (C). At different time points after biotin addition, cells were fixed, permeabilized, and stained with an antibody against the Golgi marker protein GM130, followed by detection with an Alexa568-conjugated secondary antibody. Scale bars = 20 μm. Micrographs shown here are representative of two independent experiments. **(D)** HEK 293T cells were transiently cotransfected with Sec61B-streptavidin and HLA-E_SBP_EGFP. 8 h after transfection, different concentrations of biotin were added. 24 h after transfection, cells were collected for flow cytometry analysis, and surface expression of HLA-E was assessed. MFIs shown here are representative of observations made in three independent experiments. **(E)** HEK 293T cells were transiently transfected with HLA-E_SBP_EGFP (gray area) or cotransfected with Sec61B-streptavidin and HLA-E_SBP_EGFP (red and blue lines). 8 h after transfection, biotin (50 μM final) was added to the +biotin group (red line). 24 h after transfection, cells were collected for flow cytometry analysis, and surface expression of HLA-E was assessed. MFIs shown here are representative of observations made in three experiments.

As ER retention and low surface expression of HLA-E may result from a delay in antegrade transport, we compared the trafficking of HLA-E and HLA-A3 using the RUSH (retention using selective hooks) system. In the RUSH system, HLA molecules are trapped within the ER via attachment to an ER-retained “hook” protein from which subsequent release can be triggered by biotin addition to synchronize the ER exit (Fig. 2 A; Boncompain et al., 2012). Given that isoforms of CD74, the key component of the initially designed ER hook, might be involved in HLA-I trafficking (Sugita and Brenner, 1995; Reber et al., 2002; Basha et al., 2012), we constructed an alternative hook by fusing streptavidin (str) to the N terminus of Sec61B, a type II protein localized in the ER membrane (Fig. 2 A and Fig. S2 A). The reporter protein was either HLA-E or HLA-A3 with streptavidin-binding protein (SBP) and EGFP fused to the cytoplasmic tail (Fig. 2 A).

When the hook and the reporter were cotransfected into HeLa cells, both HLA-E and HLA-A3 were localized in the ER (Fig. 2 B; and Fig. S2, B and C). After synchronous ER release by biotin addition, we tracked the antegrade transport of the reporter proteins using confocal microscopy. Within 5 min of biotin addition, both HLA-E and HLA-A3 signals were readily detectable in the Golgi, where they continued to accumulate and peaked at roughly 10–30 min, as indicated by colocalization with the cis-Golgi marker protein GM130 (Nakamura et al., 1995; Fig. 2, B–D; and Fig. S2, B and C). This signal in the Golgi subsequently declined and was almost undetectable after overnight incubation with biotin. The majority of HLA-A3 was released from the ER after biotin addition, in comparison to only a minor portion of HLA-E (Fig. 2 B and Fig. S2, B and C). Incomplete HLA-E release from the hooks was unlikely, as increasing biotin concentration did not further increase HLA-E surface expression (Fig. S2 D), and HLA-E surface expression level with or without the hook transfected was similar after overnight biotin release in HEK293T cells (Fig. S2 E).

HLA-A3 could be clearly detected at the plasma membrane within 10–15 min after ER release, and the surface signal was significant at 30 min (Fig. 2 B and Fig. S2 C). As imaging was not sensitive enough to enable robust detection of surface HLA-E, we combined flow cytometry with the RUSH system to examine the surface accumulation of HLA-E and HLA-A3. HLA-E could be detected on the plasma membrane 15 min after biotin release, and its surface expression level quickly increased until 1 h (Fig. 2 E). HLA-E then continued to accumulate at the cell surface, albeit at a lower rate, before reaching the steady-state expression level. Similar kinetics were observed for HLA-A3 upon ER release, which is consistent with our findings from confocal microscopy. Thus, the kinetics of antegrade trafficking of HLA-E and HLA-A3 appear comparable.

A proportion of HLA-A3 was detected in what appeared to be endosomal structures 1 h after biotin addition, which became more obvious after overnight biotin release (Fig. 2 B). This is in line with our observation that the surface accumulation of HLA-A3 slowed down following 1-h post-biotin addition (Fig. 2 E). Endosomal transport for HLA-E was less clear in these figures due to interference from the strong HLA-E signal in the ER. Therefore, we used live cell imaging to monitor the dynamics of HLA-E transport after ER exit. 1 h after ER release, HLA-E was detectable in the endosomal structures (Video 1). The endosomal pool increased quickly after that, which was similar to the transport patterns of HLA-A3.

**Video 1.** **Real-time imaging of the synchronized trafficking of HLA-E-SBP-EGFP.** HeLa cells were transfected to express sec61B-streptavidin as a hook and HLA-E_SBP_EGFP (green) as a reporter. 24 h after transfection, biotin (final 50 μM) was added to release HLA-E, and its trafficking was monitored by acquiring images every minute for up to 2 h using a confocal microscope. Video shown here is representative of two independent experiments. Frame rate, 4 frames/min. Scale bar = 20 μm.

Taken together, although the proportion of HLA-E and HLA-A3 molecules exiting the ER differs, they are trafficked with similar dynamics upon ER export.

### Antegrade transport of endogenous HLA-E and HLA-Ia is similar in THP1-derived macrophages

To investigate the antegrade transport of endogenous HLA-E in THP1-derived macrophages, we exposed the cells briefly to citric acid buffer to strip off surface HLA-I proteins (Ma et al., 2016; Sugawara et al., 1987) and subsequently tracked their surface recovery. Consistent with the RUSH results in HeLa cells, we observed a rapid surface recovery of both HLA-E and HLA-Ia, with around 40% of their surface expression level regained within 1 h (Fig. 2 F). Besides, there was no difference in surface recovery rate between HLA-E and HLA-Ia, although less HLA-E was released from the ER. The low surface stability of HLA-E may be the reason why it reached its steady-state surface level so quickly.

To examine the contribution of the recycling pathway to the recovery of surface proteins, we also included control samples with brefeldin A (BFA), which was added to prevent newly synthesized HLA-I molecules from reaching the cell surface (Fig. 2 F). BFA addition inhibited the recovery of both HLA-E and HLA-Ia, indicating that most surface HLA-I molecules originated from newly synthesized proteins in the ER rather than from the recycling pathway. The slight recovery of HLA-Ia in the presence of BFA could be attributed to the protein that had passed the early secretory pathway but had not yet reached the cell surface at the time of acid stripping. The further decline in the HLA-E signal following BFA addition implies that the tiny fraction of unstable HLA-E remaining on the cell surface after acid stripping was likely internalized.

To summarize, newly synthesized HLA-E and HLA-Ia are trafficked similarly to the cell surface upon ER exit in THP1-derived macrophages, which is consistent with our results obtained in HeLa cells.

### The surface stability of HLA-E is low

In addition to ER retention, low surface stability could also account for the low surface expression of HLA-E. Therefore, we incubated HeLa.E and HeLa.A3 with BFA and measured the remaining surface signal of HLA-E and HLA-A3 over time. HLA-E disappeared rapidly from the cell surface, with a half-life of less than 1 h (Fig. 2 G). On the other hand, HLA-A3 had a much longer half-life, with over 80% present at the cell surface even after 4 h. Similar results were obtained for endogenous HLA-E in THP1-derived macrophages (Fig. 2 H), which reinforces the conclusion that surface HLA-E is less stable than HLA-Ia.

### The lack of high-affinity peptides is a major factor limiting HLA-E surface expression

We then examined how the surface expression level of HLA-E is influenced by HLA-E binding peptides and β2-microglobulin (β2m), which together with HLA-E form the functional heterotrimer complex. We cotransfected a fixed amount of HLA-E plasmid with an increasing amount of plasmids encoding either β2m or different peptide minigenes into HEK293T cells and examined how these factors influence HLA-E surface expression.

**Figure 3.**
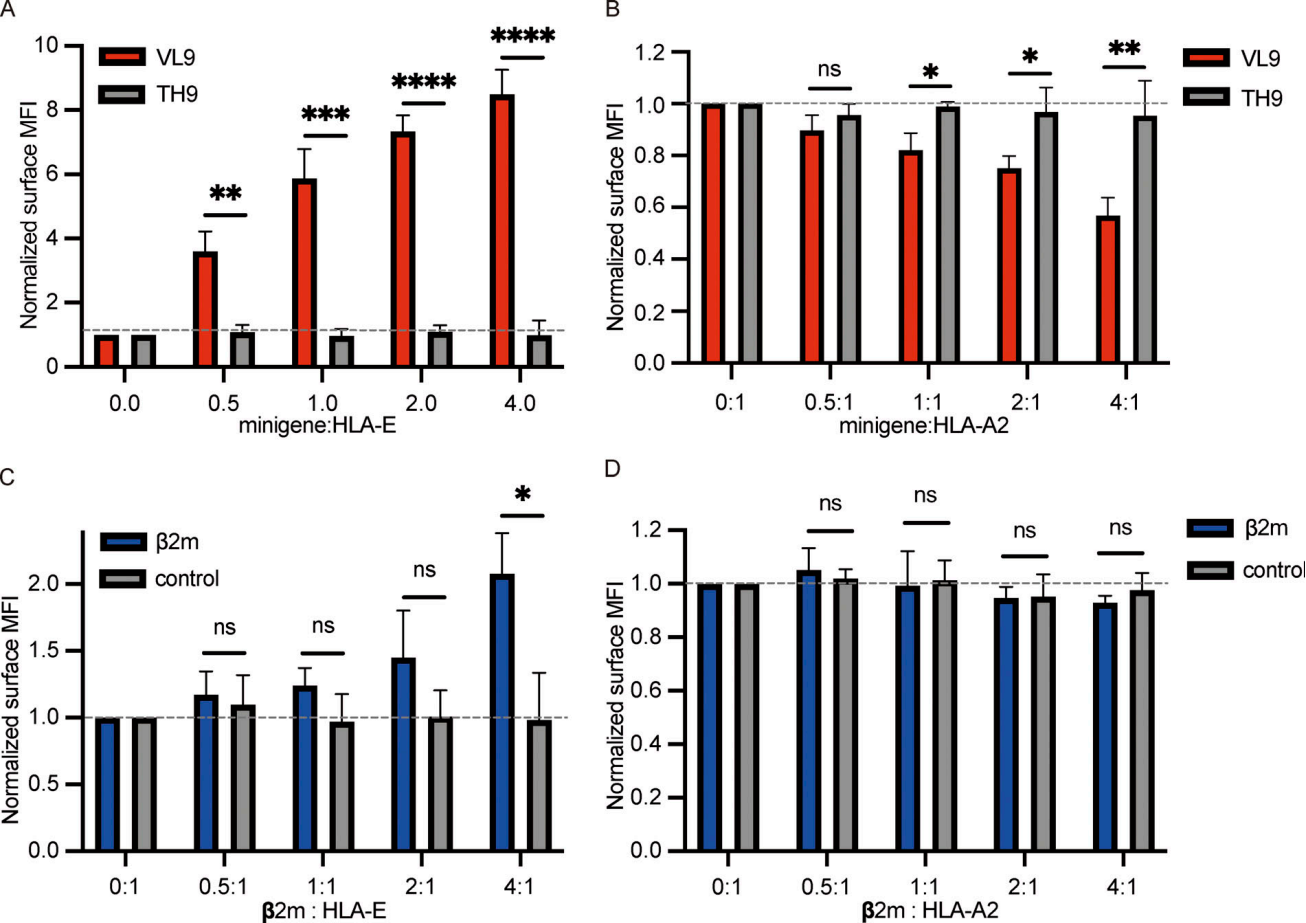
**The effect of peptides and β2m on HLA-E surface expression. (A and B)** HEK 293T cells were transiently cotransfected with HLA-E_EGFP and different peptide minigenes. **(C and D)** HEK 293T cells were transiently cotransfected with HLA-E_EGFP and β2m_EGFP or EGFP (control). The amount of HLA-E plasmid and total plasmid were the same in all samples. Expression of HLA-E and endogenous HLA-A2 were assessed. The surface level was normalized to the MFI of the sample only transfected with HLA-E_EGFP. MFIs were collected for three biological runs, and data are shown as mean ± SD (error bars). Statistical analysis was performed using unpaired two-tailed Student’s *t* test with Welch’s correction. Asterisks show the statistical significance between indicated groups: ns, not significant; *, P < 0.05; **, P < 0.01; ***, P < 0.001; ****, P < 0.0001.

VL9 (VMAPRTVLL), the canonical signal peptide that dominates HLA-E binding in normal cells (Braud et al., 1997), could efficiently promote HLA-E surface expression (Fig. 3 A), as previously reported (Lee et al., 1998). With more VL9 minigene plasmid transfected, the surface HLA-E level was augmented. When four times as much VL9 minigene plasmid was transfected as the HLA-E plasmid, we observed an eightfold increase in HLA-E surface expression. The increase in HLA-E surface expression level did not reach a plateau, implying that the lack of stably binding peptides is a major limiting step for HLA-E surface expression. The transfection of the non-HLA-E-binding TH9 peptide (TVCVIWCIH; Hannoun et al., 2018; Wu et al., 2018) did not affect the HLA-E surface level. Endogenous HLA-A2 surface expression decreased in HEK293T cells with more VL9 peptide minigene transfected (Fig. 3 B). This is possibly because the VL9 peptide enhanced HLA-E’s ability to compete for β2m by promoting the assembly of HLA-E complexes (Myers et al., 1996; Shields et al., 1999; Townsend et al., 1990).

Overexpression of β2m also enhanced HLA-E surface expression (Fig. 3 C), as illustrated in previous studies (Marín et al., 2003; Myers et al., 1996; Siddle et al., 2013; Ulbrecht et al., 1999). However, compared with VL9 peptide minigene, the effect of β2m was relatively small. We only observed a twofold increase in surface HLA-E level when the amount of β2m plasmid transfected was four times that of the HLA-E plasmid (Fig. 3 C). This could be due to β2m not being the primary limiting factor in the optimal folding of HLA-E complexes, as prior work has shown that this process is highly dependent on high-affinity peptide binding (Walters et al., 2018, 2022; Wu et al., 2018). Overexpression of β2m did not affect HLA-A2 surface expression (Fig. 3 D), which could either be due to the majority of HLA-A2 molecules being correctly folded into trimers or may be a consequence of the difference in β2m receptiveness between HLA-E and HLA-A2 (Hansen et al., 2016; Walters et al., 2018).

Therefore, while both β2m and HLA-E binding peptides regulate HLA-E surface expression, peptide availability appears to play the dominant regulatory role.

### VL9 peptide increases HLA-E surface expression mainly by promoting ER export

Given that HLA-E is enriched in the ER and is unstable on the cell surface, HLA-E binding peptides may increase its surface expression by promoting the ER export and/or surface stability of HLA-E.

**Figure 4.**
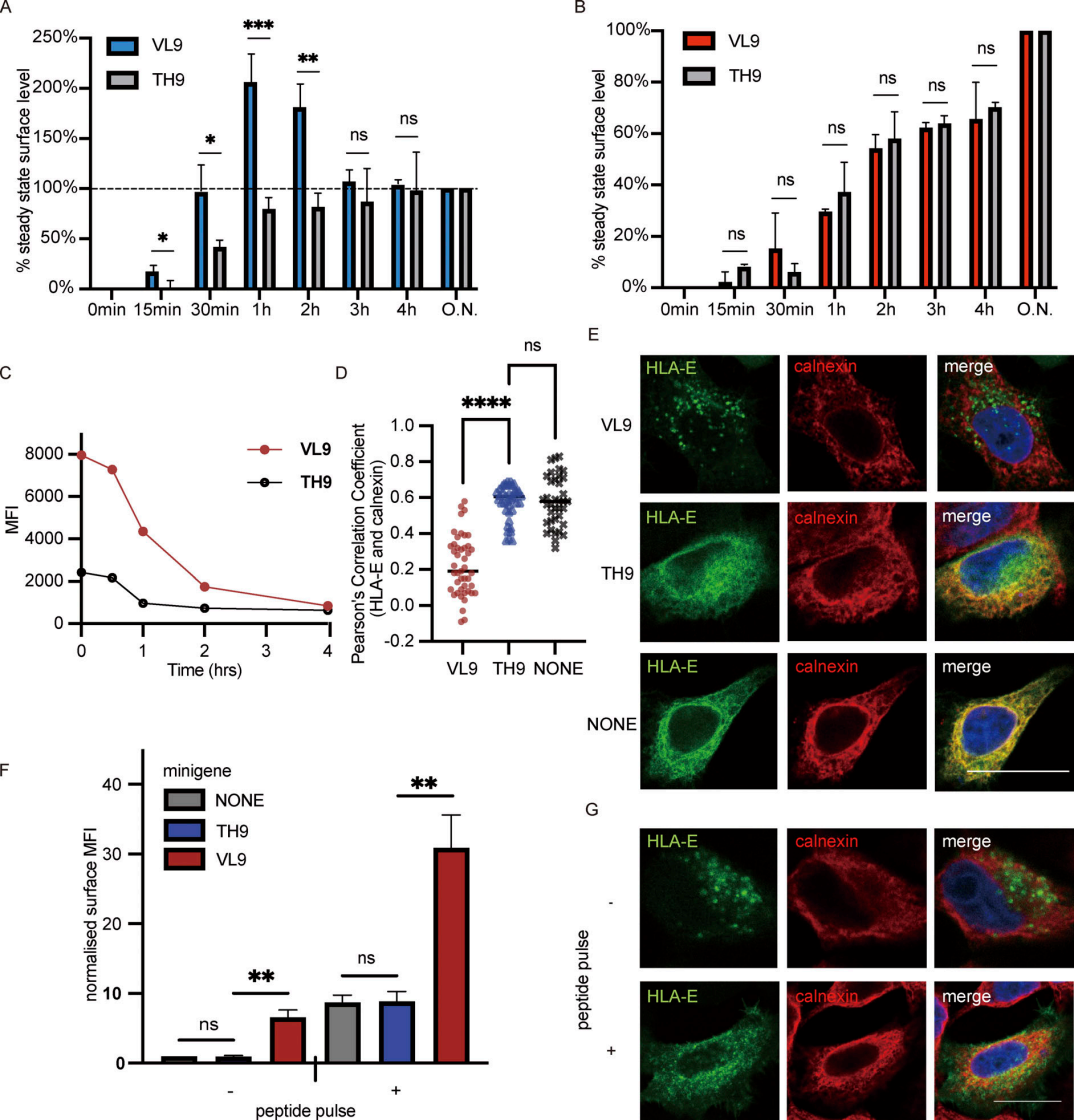
**VL9 peptide increases HLA-E surface expression mainly by promoting ER export. (A and B)** HEK 293T cells were transiently cotransfected with different peptide minigenes and the RUSH system with Sec61B-streptavidin as the hook and HLA-E_SBP_EGFP (A) or HLA-A3_SBP_EGFP (B) as the reporter protein. At different time points after biotin addition, the surface expression of HLA-E or HLA-A3 was assessed. The stable cell surface MFI after the biotin addition overnight was set to 100%, the MFI before biotin addition was set to 0%, and the other values were normalized accordingly. Data were collected for three biological runs and are shown as mean ± SD (error bars). **(C)** HEK 293T cells transiently cotransfected with HLA-E_EGFP and different peptide minigenes were incubated in media containing BFA for different time points, and surface expression of HLA-E molecules was assessed. The average cell surface MFIs are shown, and the results are representative of observations made in three experiments. **(D and E)** HeLa cells were transiently transfected with HLA-E_EGFP or co-transfected with HLA-E_EGFP and different peptide minigenes. Cells were fixed, permeabilized, and stained with an antibody against the ER marker protein calnexin, followed by detection with an Alexa647-conjugated secondary antibody. **(D)** Quantification of ER colocalization. PCC was calculated for 30–50 individual cells, and the PCC values of each cell and the mean values are shown. **(E)** Representative confocal micrographs of different conditions. Scale bar = 20 μm. Micrographs shown here are representative of two independent experiments. **(F)** HEK 293T cells were transiently cotransfected with HLA-E_EGFP and different peptide minigenes. 8 h after transfection, VL9 peptide (100 μM final) was added to the peptide pulse–positive group, and an equal amount of DMSO was added to the peptide pulse–negative group as the control. 24 h after transfection, cells were collected for flow cytometry analysis. The surface expression of HLA-E was normalized to the MFI of the control group with neither minigene transfection nor peptide pulse treatment. **(G)** Representative micrographs of HeLa cells transiently transfected with HLA-E_EGFP and VL9 peptide minigene. 8 h after transfection, VL9 peptide (100 nM) was added to the peptide pulse–positive group, and an equal amount of DMSO was added to the peptide pulse–negative group as the control. 24 h after transfection, cells were fixed, permeabilized, and stained with an antibody against the ER marker protein calnexin, followed by detection with an Alexa647-conjugated secondary antibody. Scale bar = 20 μm. Micrographs shown here are representative of two independent experiments. Statistical analysis was performed using unpaired two-tailed Student’s *t* test with Welch’s correction (A and B) or one-way ANOVA with Tukey’s post-hoc test (D and F). Asterisks show the statistical significance between indicated groups: ns, not significant; *, P < 0.05; **, P < 0.01; ***, P < 0.001; ****, P < 0.0001.

To investigate the role of HLA-E binding peptides, we cotransfected the RUSH system with different peptide minigenes into HEK293T cells. In the presence of the VL9 peptide minigene, we observed a rapid increase of surface HLA-E signal after biotin addition (Fig. 4 A), confirming that VL9 was potent in releasing HLA-E ER retention. This is consistent with previous findings that the VL9 peptide facilitates the optimal folding of HLA-E complexes (Walters et al., 2018, 2022; Wu et al., 2018), a process crucial for ER export. In comparison, the surface HLA-E signal accumulated much more slowly when cotransfected with the control TH9 peptide minigene. The large amount of HLA-E simultaneously driven to the cell surface by VL9 was not stable and was internalized quickly, as the HLA-E surface level started to decline after peaking 1 h after ER release. This is probably because even though VL9 is currently one of the best-known HLA-E binding peptides, its binding is modest compared with that of the very strongest HLA-Ia binding peptides (Barber et al., 2022; Hellman et al., 2016; Walters et al., 2022). In the presence of the TH9 peptide minigene, the surface accumulation of the HLA-E signal reached a plateau 1 h after biotin addition. In contrast, surface HLA-A3 continued to increase 4 h after ER release before it stabilized (Fig. 4 B). This is in line with the finding that surface HLA-A3 is more stable than HLA-E. Taken together, these results indicate that endogenous VL9 peptide expression can productively release HLA-E from the ER but does not effectively stabilize surface HLA-E.

To further characterize how HLA-E binding peptides affect its surface stability, a BFA decay assay was performed. We cotransfected HEK293T cells with HLA-E and different peptide minigenes and assessed the surface HLA-E level following various BFA incubation times (Fig. 4 C). While the VL9 minigene significantly boosted HLA-E surface expression compared with the non-binder TH9, it did not significantly enhance HLA-E surface stability, and a rapid drop in surface HLA-E level was observed after BFA addition (Fig. 4 C).

**Figure S3.**
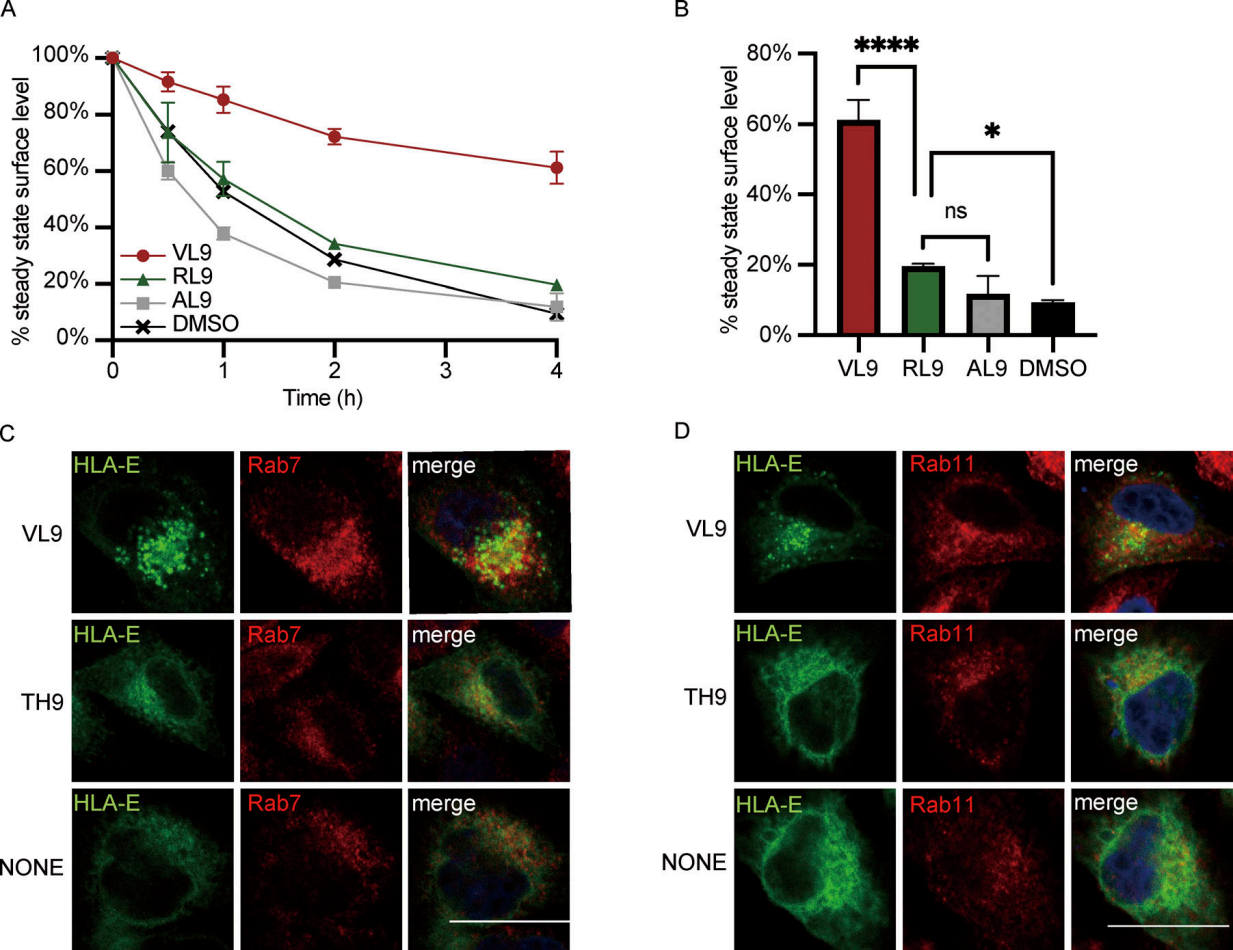
**Functions of HLA-E peptides. (A and B)** K562E cells were first incubated in media containing different peptides for 3 h before BFA addition. After BFA incubation for different time points, the surface expression of HLA-E molecules was assessed. **(A)** The average cell surface MFI for time point zero of each sample was set to 100% and the following values were normalized as its percentage. **(B)** Percentage of surface HLA-E after BFA incubation for 4 h. MFIs were collected and plotted for three biological runs, and data are shown as mean ± SD (error bars). Statistical analysis was performed using one-way ANOVA with Tukey’s post-hoc test. Asterisks show the statistical significance between indicated groups: ns, not significant; *, P < 0.05; ****, P < 0.0001. **(C and D)** Representative micrographs of HeLa cells transiently transfected with HLA-E_EGFP or cotransfected with HLA-E_EGFP and different peptide minigenes. Cells were fixed, permeabilized, and stained with antibodies against protein markers of the late endosome (Rab7; C) or the recycling endosome (Rab11; D). Cells were then stained with Alexa647-conjugated secondary antibody. Scale bar = 20 μm. Micrographs shown here are representative of two independent experiments.

We further validated the function of the endogenous VL9 peptide via confocal microscopy. Cotransfection of the VL9 peptide minigene alleviated HLA-E ER retention (Fig. 4, D and E). When cotransfected with the TH9 peptide minigene, the majority of HLA-E remained in the ER, resembling its distribution without minigene cotransfection. Despite the potent ER-releasing effect of the VL9 peptide minigene, the HLA-E surface signal was almost undetectable via confocal microscopy (Fig. 4 E). However, when an excess of exogenous VL9 peptide was also added, which could increase the surface stability of HLA-E (Fig. S3, A and B), we saw an obvious surface accumulation of HLA-E (Fig. 4, F and G). This reinforces our conclusion that the main mechanism by which endogenous VL9 increases HLA-E surface expression is not the improvement of its surface stability as exogenous VL9 peptide pulsing and endogenous VL9 peptide minigene expression had a synergistic effect on boosting HLA-E surface expression.

In conclusion, HLA-E binding peptides like VL9 increase HLA-E surface expression primarily by promoting ER exit but do not enhance its surface stability under physiological conditions.

### VL9 peptide–driven ER egress promotes HLA-E accumulation in early endosomes and lysosomes

**Figure 5.**
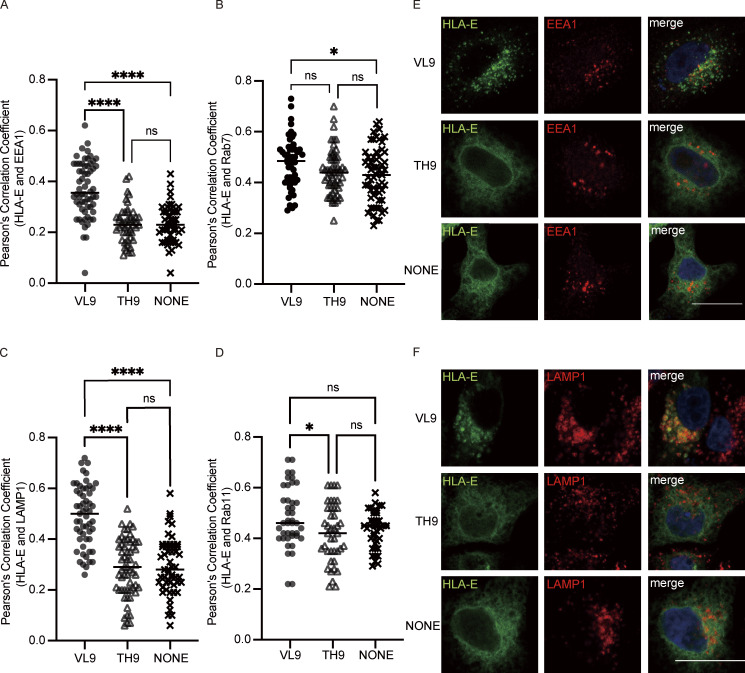
**VL9 peptide enriches HLA-E in early endosomes and lysosomes.** HeLa cells were transiently transfected with HLA-E_EGFP or cotransfected with HLA-E_EGFP and different peptide minigenes. Cells were fixed, permeabilized, and stained with antibodies against protein markers of the early endosome (EEA1), late endosome (Rab7), lysosome (LAMP1), or recycling endosome (Rab11). Cells were then stained with Alexa647-conjugated secondary antibody. **(A–D)** Quantification of colocalization of HLA-E with different marker proteins. The PCC values of each cell and the mean values are shown with 30–60 cells per sample. Statistical analysis was performed using one-way ANOVA with Tukey’s post-hoc test. Asterisks show the statistical significance between indicated groups: ns, not significant; *, P < 0.05; ****, P < 0.0001. **(E and F)** Representative confocal micrographs of HLA-E colocalizing with early endosome (E) or lysosome (F) under different conditions. Scale bar = 20 μm. Data shown are representative of two independent experiments.

As the surface stability of HLA-E molecules driven out of ER via endogenous VL9 peptide expression was not improved, we hypothesized that they likely ended up in endosomes. Therefore, we cotransfected different peptide minigenes and HLA-E into HeLa cells and used confocal microscopy to investigate the endosomal distribution of HLA-E. The cotransfection of VL9 peptide minigene led to the enrichment of HLA-E in early endosomes (Fig. 5, A and E) and lysosomes (Fig. 5, C and F). Meanwhile, no additional HLA-E accumulation was observed in late endosomes (Fig. 5 B and Fig. S3 C) or recycling endosomes (Fig. 5 D and Fig. S3 D). In comparison, coexpression of the TH9 peptide did not alter the endosomal distribution of HLA-E.

### The cytoplasmic tail of HLA-E leads to intracellular accumulation of HLA-I

**Figure 6.**
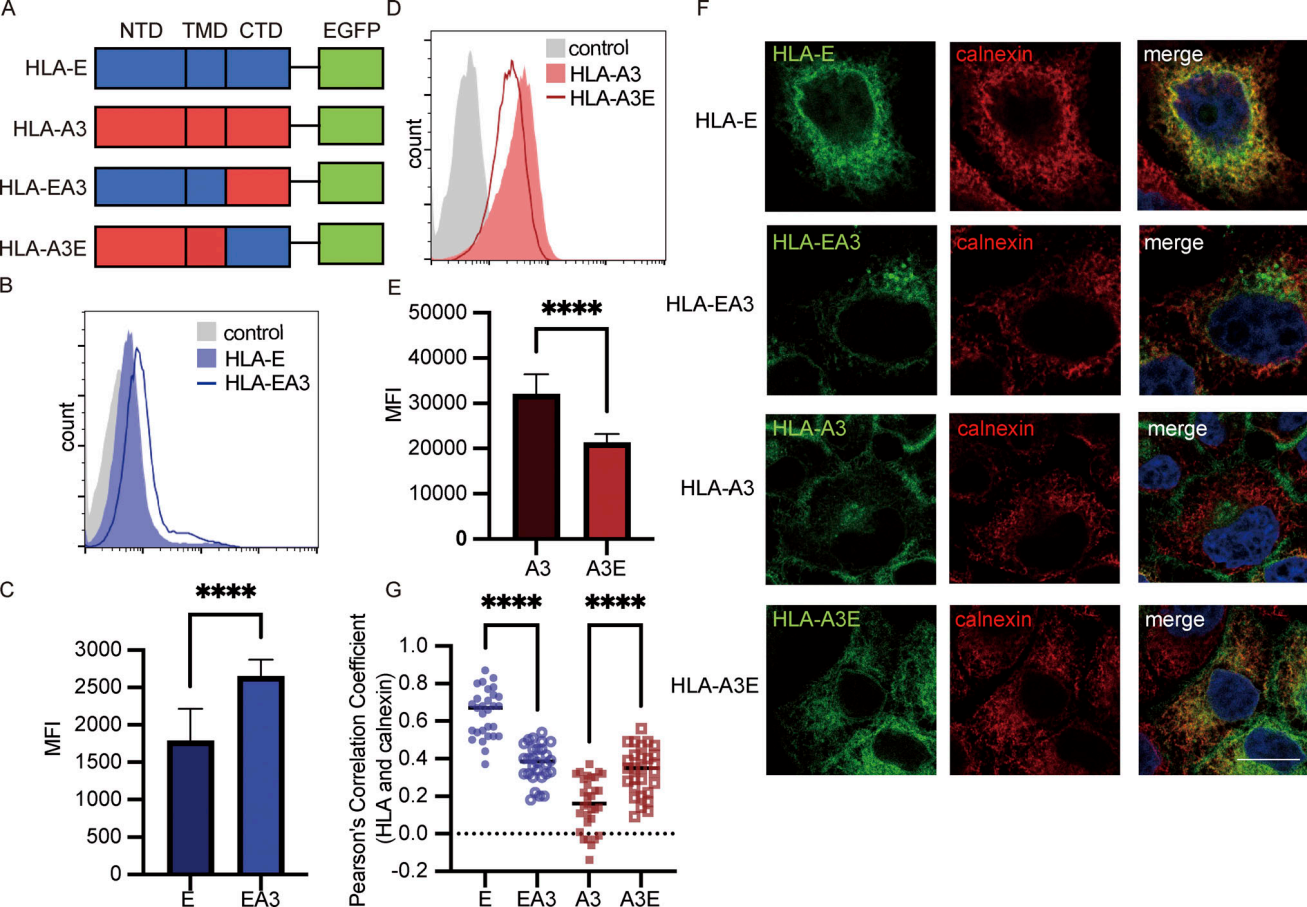
**HLA-E cytoplasmic tail contributes to intracellular accumulation. (A)** Schematic representation of different HLA constructs. HLA-EA3 has the N-terminal domain (NTD) and transmembrane domain (TMD) of HLA-E and the cytoplasmic tail (CTD) of HLA-A3. HLA-A3E has the NTD and TMD of HLA-A3 and the CTD of HLA-A3. All constructs were tagged with EGFP on the C terminus. **(B–E)** HeLa cells stably expressing different HLA molecules were collected for flow cytometry analysis. **(B)** Representative graph of surface MFI of HLA-E (light blue area) and HLA-EA3 (dark blue line). **(D)** Representative graph of surface MFI of HLA-A3 (light red area) or HLA-A3E (dark red line). MFI of the unstained sample (gray area) was used as the negative control. MFIs shown here are representative of observations made in six experiments. **(C and E)** MFIs were collected and plotted for six biological runs, and data are shown as mean ± SD (error bars). **(F and G)** HeLa cells stably expressing different HLA molecules were fixed, permeabilized, and stained with an antibody against the ER marker protein calnexin, followed by detection with an Alexa568-conjugated secondary antibody. **(F)** Representative confocal micrographs of HeLa cells stably expressing different HLA molecules. Scale bar = 20 μm. Micrographs shown here are representative of two independent experiments. **(G)** Quantification of colocalization of different constructs with the ER marker protein calnexin. The PCC values of each cell and the mean values are shown with 20–40 cells per sample. Statistical analysis was performed using paired two-tailed Student’s *t* test (C and E) or unpaired two-tailed Student’s *t* test with Welch’s correction (G). Asterisks show the statistical significance between indicated groups: ****, P < 0.0001.

Previous studies have shown that the cytoplasmic tail of MHC-I molecules influences endocytosis (Vega and Strominger, 1989), degradation (Park et al., 2001), and ER retrieval (Boyle et al., 2006; Cho et al., 2011). To investigate if the HLA-E cytoplasmic tail plays a part in shaping its unique transport, we swapped the cytoplasmic domains of HLA-E and HLA-A3 to construct two chimeric proteins HLA-EA3 and HLA-A3E (Fig. 6 A) and generated HeLa cell lines stably expressing these two constructs.

**Figure S4.**
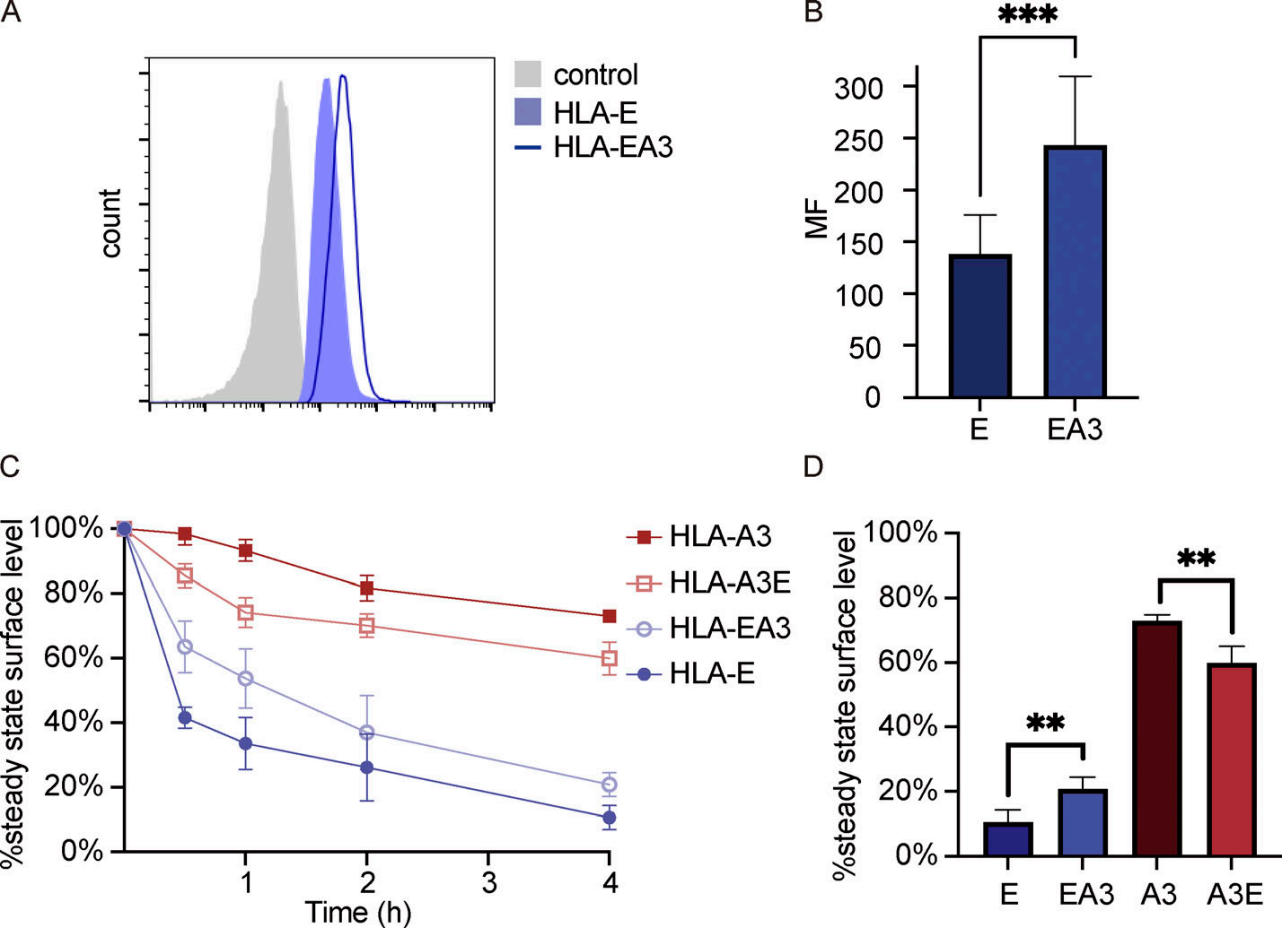
**EGFP tagging does not seem to significantly affect the function of HLA-E cytoplasmic tail. (A and B)** HEK293T cells were transiently cotransfected with EGFP and HLA-E or HLA-EA3 without EGFP tagging. 24 h after transfection, cells were collected for flow cytometry analysis (gated on GFP^+^ cells). Expression of HLA molecules was assessed with anti-HLA-E antibody (3D12). **(A)** Representative graph of surface MFI of HLA-E (light blue area) and HLA-EA3 (dark blue line). MFI of unstained samples (gray area) was used as the negative control. MFI shown here is representative of the observations made in five experiments. **(B)** MFI were collected and plotted for five biological runs, and data were shown as mean ± SD (error bars). **(C and D)** HEK293T cells were transiently cotransfected with EGFP and different HLA constructs without EGFP tagging. 24 h after transfection, cells were incubated in media containing BFA for different time points, and the surface expression of HLA molecules was assessed. **(C)** The average cell surface MFI for time point zero was set to 100% and the following values were normalized as its percentage. **(D)** The percentage of HLA on the cell surface after BFA incubation for 4 h. Data were collected for six biological runs and are shown as mean ± SD (error bars). Statistical analysis was performed using paired (B) or unpaired (D) two-tailed Student’s *t* test. Asterisks show the statistical significance between indicated groups: **, P < 0.01; ***, P < 0.001.

HLA-EA3 surface expression was ∼1.7-fold of that of HLA-E (Fig. 6, B and C), and HLA-A3E surface expression was about 0.7-fold of that of HLA-A3 (Fig. 6, D and E), as evaluated by flow cytometry. We also compared the surface expression level of HLA-EA3 and HLA-E without EGFP tagging and observed similar results (Fig. S4, A and B), suggesting that EGFP tagging at the cytoplasmic tail did not distort its function in regulating protein transport. This moderate downregulating effect of the HLA-E cytoplasmic tail was further confirmed through confocal microscopy (Fig. 6 F). Compared with HLA-A3, HLA-A3E showed a lower surface signal and a higher intracellular signal. We did not observe an obvious surface signal for HLA-EA3 potentially because the HLA-EA3 surface level was low despite being higher than that of HLA-E, as most HLA-EA3 stayed intracellularly (Fig. 6 F). Collectively, these results show that the HLA-E cytoplasmic tail likely contributes to its low surface expression.

As most HLA-E was retained in the ER, we then examined if the HLA-A3 cytoplasmic tail could improve HLA-E ER egress. Compared with HLA-A3, HLA-A3E had a greater ER colocalization (Fig. 6, F and G). In HLA-EA3–expressing cells, we noted a decrease in ER retention compared with HLA-E. Therefore, the HLA-E cytoplasmic tail contributes to its ER retention. The swapping of the cytoplasmic tails did not completely reverse the ER retention level, which is consistent with our findings that other factors, particularly the shortage of high-affinity peptides, also regulate the ER exit of HLA-E.

Taken together, these results indicate that the HLA-E cytoplasmic tail downregulates HLA-I surface expression and facilitates ER retention, although the effect appears minor compared with peptide supply.

### The fast internalization of HLA-E mainly relies on its cytoplasmic tail

**Figure 7.**
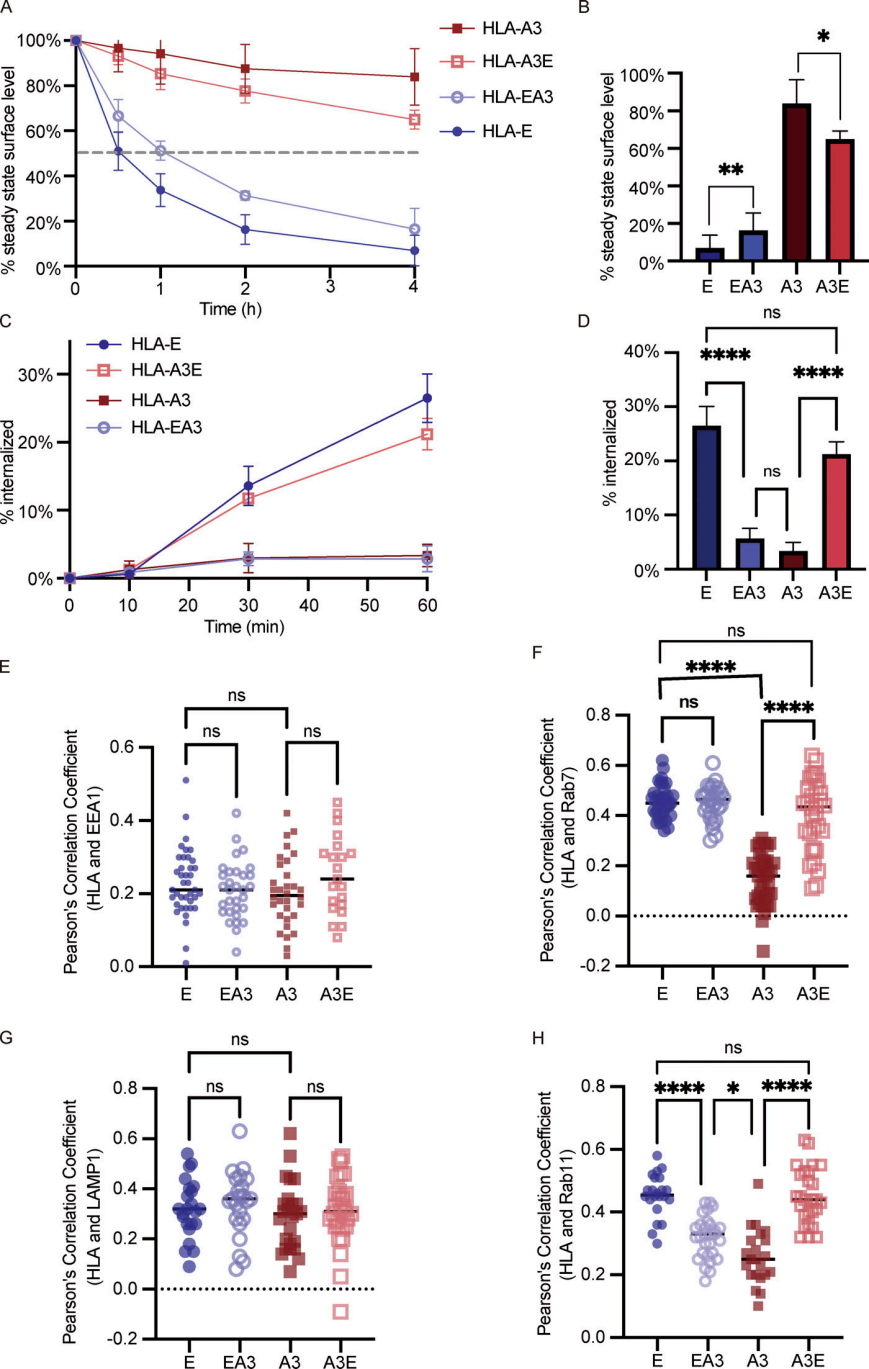
**HLA-E cytoplasmic tail facilitates internalization and endosomal enrichment. (A and B)** HeLa cells stably expressing different HLA constructs were incubated in media containing BFA for different time points, and surface expression of HLA molecules was assessed. **(A)** The average cell surface MFI for time point zero was set to 100% and the following values were normalized as their percentage. The dashed line represents 50%. **(B)** The percentage of HLA on the cell surface after BFA incubation for 4 h. Data were collected for four biological runs and are shown as mean ± SD (error bars). **(C and D)** Surface HLA molecules of HeLa stable cell lines were labeled, and then the cells were incubated in media containing primaquine. After different time points of internalization, samples were collected, and uninternalized surface antibody–HLA complexes were stripped off using citric acid. **(C)** The MFI of antibody-labeled cells without acid stripping was set to 100%, and the MFI of antibody-labeled cells with acid stripping but without internalization was set to 0%. The percentage of internalization was quantified by the normalization of MFI increase accordingly. **(D)** The percentage of HLA internalized after 1 h. Data were collected for four biological runs and are shown as mean ± SD (error bars). **(E–H)** HeLa cells stably expressing different HLA constructs were fixed, permeabilized, and stained with antibodies against protein markers for early endosome (EEA1; E), late endosome (Rab7; F), lysosome (LAMP1; G), or recycling endosome (Rab11; H). Cells were then stained with Alexa568-conjugated secondary antibody. The colocalization of HLA-E, HLA-EA3, HLA-A3, and HLA-A3E with different endosomal compartment markers was quantified using PCC, with 20–40 cells per sample. The PCC values of each cell and the mean values are shown. Micrographs shown here are representative of two independent experiments. Statistical analysis was performed using one-way ANOVA with Tukey’s post-hoc test. Asterisks show the statistical significance between indicated groups: ns, not significant; *, P < 0.05; **, P < 0.01; ****, P < 0.0001.

After determining the effect of the cytoplasmic tail on ER export and surface expression level, we explored how the cytoplasmic tail influences the surface turnover of HLA molecules. HLA-E cytoplasmic tail shortened the surface half-life of HLA-I molecules compared with their counterparts with the HLA-A3 cytoplasmic tail (Fig. 7, A and B). The half-life of surface HLA-E was around 30 min compared with 1 h for HLA-EA3. Less than 20% of HLA-A3 was endocytosed after 4 h, while more than 30% of HLA-A3E was endocytosed in the same time frame (Fig. 7, A and B). Therefore, the cytoplasmic tail of HLA-E contributes to its low surface stability. However, the HLA-A3 cytoplasmic tail only effectively stabilized HLA-EA3 at early time points, and HLA-EA3 was much more unstable than HLA-A3, suggesting that the cytoplasmic tail might not be the major determinant of the surface stability of HLA-I molecules. Given that EGFP tagging might obstruct interactions crucial for the regulation of endocytosis, we also compared the surface stability of different constructs without EGFP tagging (Fig. S4, C and D). The similar results observed further implied that the regulatory functions of the cytoplasmic tail on endocytosis are not discernibly affected by the addition of the C-terminal tag we used.

**Figure S5.**
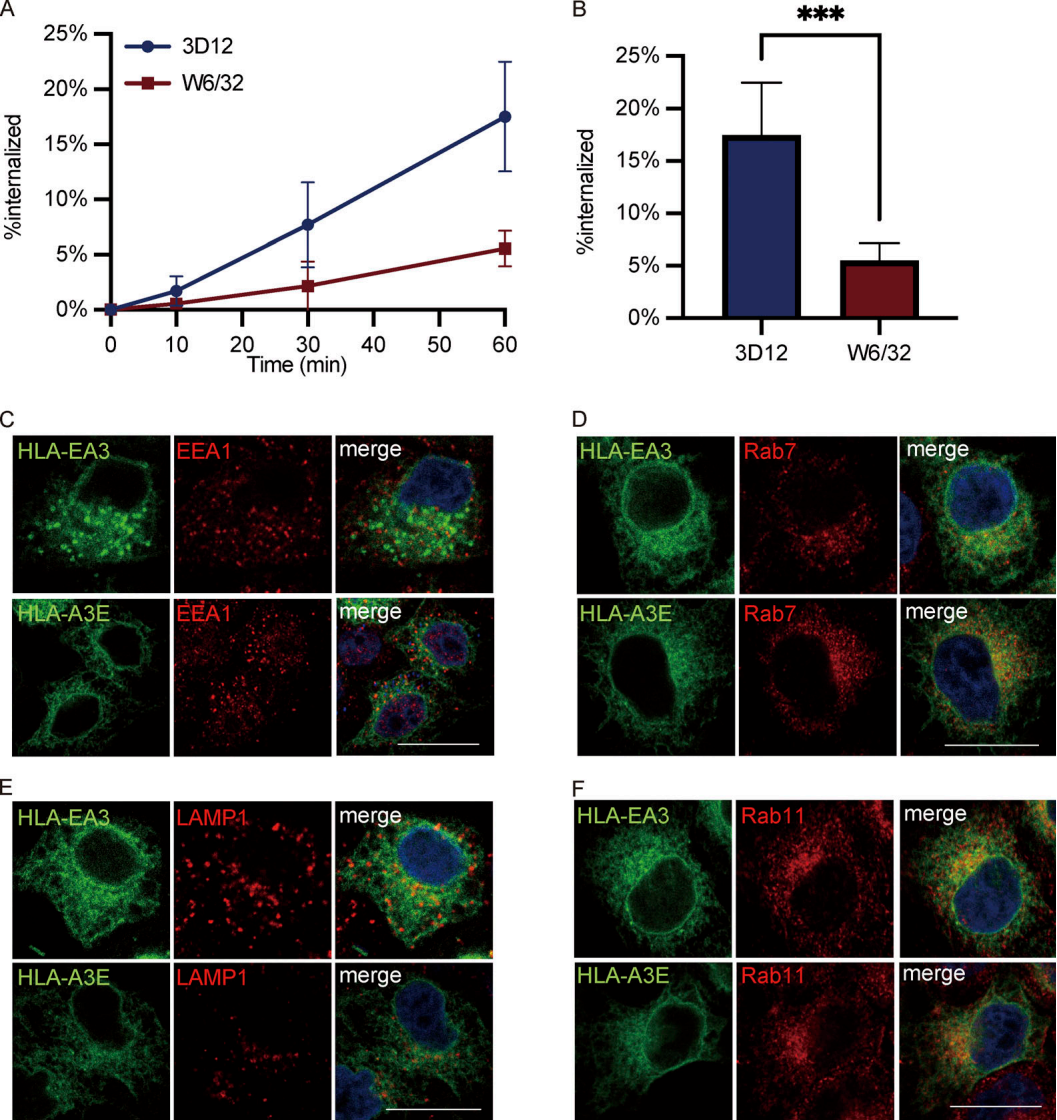
**Functions of HLA-E cytoplasmic tail on internalization and endosomal distribution. (A and B)** Surface HLA molecules of THP1-differentiated macrophages were labeled, and then the cells were incubated in media containing primaquine. After different time points of internalization, samples were collected, and uninternalized surface antibody–HLA complexes were stripped off using citric acid. **(A)** The MFI of antibody-labeled cells without acid stripping was set to 100%, and the MFI of antibody-labeled cells with acid stripping but without internalization was set to 0%. The percentage of internalization was quantified by the normalization of MFI increase accordingly. **(B)** The percentage of HLA-E or HLA-Ia molecules internalized after 1 h. Data were collected for three biological runs and are shown as mean ± SD (error bars). Statistical analysis was performed using unpaired two-tailed Student’s *t* test with Welch’s correction. Asterisks show the statistical significance between indicated groups: *, P < 0.05. **(C–F)** Representative micrographs of HeLa cells stably expressing HLA-EA3 or HLA-A3E. After fixation and permeabilization, cells were stained with antibodies against protein markers for early endosome (EEA1; C), late endosome (Rab7; D), lysosome (LAMP1; E), and recycling endosome (Rab11; F), followed by detection with Alexa568-conjugated secondary antibody. Scale bars = 20 μm. Micrographs shown here are representative of two independent experiments.

To further explore if the cytoplasmic tail regulates internalization, we employed an acid-stripping assay to measure the internalization rate of surface HLA molecules as previously described (Ma et al., 2016). After surface labeling, surface HLA molecules from different HeLa cell lines were allowed to internalize for different lengths of time, followed by acid stripping to remove the remaining surface proteins. Rapid internalization could be observed for both HLA-E and HLA-A3E, with over 20% internalized within 1 h (Fig. 7, C and D). In contrast, the internalization of HLA-A3 and HLA-EA3 was much slower, with <5% internalized within 1 h. The fast internalization of surface HLA-E was also observed in THP1-derived macrophages (Fig. S5, A and B). The cytoplasmic tail of HLA-E seems to be the main regulator of its fast surface internalization as tail swapping almost completely reversed the internalization rate of HLA-E and HLA-A3 (Fig. 7, C and D).

Moreover, we found that the difference in internalization rate introduced by the cytoplasmic tail appears to dictate some of the difference in surface stability, at least at early time points. 1 h after BFA addition, 20% more HLA-E disappeared from the cell surface compared with HLA-EA3 (Fig. 7, A and B). Simultaneously, 20% more surface HLA-E was internalized after 1 h (Fig. 7, C and D). Compared with HLA-A3, about 15% more HLA-A3E disappeared from the cell surface 1 h after BFA addition (Fig. 7, A and B), whereas a comparable proportion of HLA-A3E was internalized (Fig. 7, C and D). Therefore, quicker internalization appears to be the main cause of the decreased surface expression introduced by the HLA-E cytoplasmic tail.

### HLA-E cytoplasmic tail contributes to its enrichment in late endosomes and recycling endosomes

As the HLA-E cytoplasmic tail regulates endocytosis, we then investigated whether it affects the distribution of HLA-I molecules in different endosomal compartments. While HLA-E was more enriched in late endosomes and recycling endosomes than HLA-A3 in HeLa cells (Fig. 1, E, G, and H), there was no difference in terms of enrichment in either of these compartments between HLA-E and HLA-A3E (Fig. 7, F and H; and Fig. S5, D and F). This indicates that the HLA-E cytoplasmic tail could influence the endosomal distribution of HLA-I molecules.

However, swapping the cytoplasmic tail did not completely reverse the endosomal distribution of HLA molecules. While HLA-EA3 was less enriched in recycling endosomes compared with HLA-E, the late endosomal enrichment was retained, implying that factors other than the cytoplasmic tail also contribute to the enrichment of HLA-E in late endosomes. The slight difference between HLA-EA3 and HLA-A3 in the enrichment of recycling endosomes also suggests the existence of other regulatory factors. No difference was observed in the distribution of these different constructs in early endosomes and lysosomes (Fig. 7, E and G; and Fig. S5, C and E). Taken together, these results show that the HLA-E cytoplasmic tail is important in shaping the endosomal distribution of HLA-I molecules, contributing to their enrichment in late endosomes and recycling endosomes.

## Discussion

MHC-E–restricted CD8^+^ T cells have been reported in different infections. HLA-E–restricted CD8^+^ T cells are a prominent component of the immune response against Mtb in humans (Caccamo et al., 2015; Heinzel et al., 2002; Prezzemolo et al., 2018; van Meijgaarden et al., 2015) and are associated with the protective effect of a vaccine against *S. typhi* (Rudolph et al., 2019; Salerno-Gonçalves et al., 2004). Vaccine-elicited MHC-E–restricted CD8^+^ T cells can also eradicate SIV in the initial stages of acute infection in RMs (Hansen et al., 2016). The widespread expression, low polymorphism, and relative resistance to downregulation by pathogens make HLA-E a novel and highly promising target for immunotherapies and vaccine design. However, a big obstacle to the design of vaccines that will elicit effective HLA-E–restricted CD8^+^ T cell responses is the poor understanding of HLA-E transport and pathogen peptide loading. Here, we made a detailed characterization of the dynamic process of HLA-E trafficking and identified two key factors that regulate HLA-E surface expression. The first was a shortage of high-affinity peptides that led to ER retention and low surface stability, which together resulted in low surface expression of HLA-E. The second was the HLA-E cytoplasmic tail, which not only further facilitated its ER retention but also was the main determining factor for its fast internalization from the cell surface.

Previous studies have shown that the VL9 peptide is required for HLA-E surface expression (Braud et al., 1997, 1998b). Although the VL9 peptide is universally expressed, we found that HLA-E was not saturated by endogenous VL9, as overexpression of the VL9 peptide via minigene transfection significantly increased the surface HLA-E level (Fig. 3). The oversupply of HLA-E probably ensures its effectiveness in monitoring HLA-Ia expression and regulating NK cell activity (Strong et al., 2003).

Compared with the binding groove of HLA-Ia molecules, specific amino acids at positions 67, 143, 147, 152, and 156 in HLA-E optimize VL9 binding (O’Callaghan et al., 1998), thus leading to a VL9-dominated peptidome of HLA-E. In parallel, the peptidome of the murine MHC-E orthologue Qa-1 is dominated by the Qdm peptide, which is derived from the MHC-Ia leader sequence (Kambayashi et al., 2004). Structural studies have shown that HLA-E refolds into a compact, fully folded form when bound to VL9 (Walters et al., 2018; Walters et al., 2022), which is possibly essential for HLA-E to pass the potential proofreading of tapasin and TAP-binding protein-related necessary for ER exit (Thomas and Tampé, 2019). This is consistent with our observation here that the expression of the VL9 minigene was potent in releasing HLA-E from the ER (Fig. 4). Low-affinity peptides do not stabilize HLA-E well (Walters et al., 2018; Walters et al., 2022), which may explain why they fail to promote efficient ER export of HLA-E (Thomas and Tampé, 2019). The favored binding of VL9 serves HLA-E’s primary function of presenting VL9 to NKG2-CD94 receptors to indicate normal expression of classical HLA-Ia molecules.

Although VL9 is the strongest known HLA-E binding peptide, its affinity for HLA-E is low compared with the average affinity of most HLA-Ia binding peptides (Hoare et al., 2008; Strong et al., 2003; Walters et al., 2022), leading to a short half-life of HLA-E-VL9–bound complexes in vitro (Barber et al., 2022). This is in accordance with our finding that endogenous VL9 did not stabilize the long-term expression of HLA-E on the cell surface under physiological conditions (Fig. 4). Similarly, Qa1 fails to form stable complexes with its optimal binding peptide Qdm (Kambayashi et al., 2004). This enables MHC-E to provide an “up-to-date,” dynamic picture of cellular MHC-I availability, thus allowing timely NK recognition of “unhealthy” cells.

In contrast to HLA-Ia molecules, empty HLA-E can retain its association with β2m and is peptide receptive, at least in vitro (Walters et al., 2018). This unusual property suggests that HLA-E could acquire new peptides from endosomal compartments after internalization. Alternative endosomal antigen processing pathways of HLA-E for mycobacteria-derived peptides in macrophage phagolysosomes have been described (Grotzke et al., 2009). In the RhCMV/SIV vaccine, MHC-E–restricted antigen presentation is independent of TAP, and MHC-E is enriched in endosomal structures (Verweij et al., 2021). Based on these studies, we suggest that the HLA-E cytoplasmic tail, which could relocate HLA-I molecules to late endosomes and the recycling endosomes (Fig. 7), may be crucial for the presentation of some pathogen-derived peptides by HLA-E.

Our findings shed light on the potential mechanisms by which HLA-E is relatively resistant to surface downregulation during infection. Inhibition of ER export is a strategy commonly employed by pathogens to downregulate surface HLA-Ia molecules. This is often accomplished by inhibiting the functions of the proteasome, TAP, or other associated chaperones to block efficient peptide loading (Hansen and Bouvier, 2009). As HLA-E is already stuck in the ER mainly due to the lack of high-affinity peptides, this method might be ineffective in further downregulating its surface level. Many pathogens can also promote the internalization of surface MHC-Ia molecules by targeting the cytoplasmic tail, including HIV-1 (Cohen et al., 1999; Le Gall et al., 1998; Roeth et al., 2004; Williams et al., 2002; Wonderlich et al., 2008), EBV (Griffin et al., 2013), and Kaposi’s sarcoma–associated herpesvirus (Coscoy et al., 2001; Hewitt et al., 2002). Many cancer cells also target the cytoplasmic tail of MHC-I to modulate its intracellular distribution (Bradley et al., 2015; Wang et al., 2008). As HLA-E already undergoes rapid internalization due to its cytoplasmic tail (Fig. 7), the efficacy of this approach is likely to be low. Unidentified motifs in the HLA-E cytoplasmic tail might also contribute to its resistance to surface downregulation. In addition, pathogens can provide HLA-E binding peptides to further enhance HLA-E surface expression and evade NK cell detection (Sharpe et al., 2019). Furthermore, since atypical antigen processing has been reported for HLA-E, other mechanisms may also exist to further promote the alternative transport of HLA-E to maintain its surface expression level.

Natural MHC-E–restricted T cell priming by HIV or SIV is rare, which is possibly due to the limited amount of HLA-E available at the surface of most cells (Hansen et al., 2016). The TAP-independent MHC-E transport, promoted by the provision of excessive VL9 peptide from Rh67 or UL40, is crucial for priming the efficacious MHC-E–restricted CD8^+^ T cell response in the RhCMV/SIV vaccine (Verweij et al., 2021). UL40 and Rh67 lead to the enrichment of HLA-E in endosomal structures (Verweij et al., 2021), which is compatible with our findings here (Fig. 5). Such endosomal enrichment seems to be largely due to the moderate binding affinity of VL9, which allows for potent ER export of HLA-E but is not sufficient to confer a long surface half-life of HLA-E (Fig. 4). With more MHC-E in the endosomal pathway, the presentation of pathogen-derived peptides may be more efficient, thus facilitating the priming of MHC-E–restricted CD8^+^ T cells. Increasing the amount of HLA-E in the endosomal pathway might be crucial for the development of vaccines aimed at inducing potent HLA-E–restricted CD8^+^ T cell responses. This may be achieved by a combination of strategies, including downregulating competing classically restricted T cell responses and driving more HLA-E out of ER through the overexpression of endogenous VL9 peptides.

While we delineated an overall picture of HLA-E transport and defined some key mechanistic determinants thereof, some points remain unresolved. Surface murine MHC-Ia molecules with different conformations undergo different transport pathways upon endocytosis (Mahmutefendić et al., 2011; Mahmutefendić et al., 2013; Zagorac et al., 2012). Given the similarity between MHC-Ia open conformers and HLA-E, it will be important to further explore and compare the trafficking of HLA-E of different conformations. Although the HLA-E/VL9 complex had a short half-life on the cell surface, it is unclear to what extent the VL9 peptide is lost primarily at the plasma membrane. If HLA-E is internalized in an empty β2m-associated but peptide-receptive form, future research should address where and how HLA-E acquires new peptides and gets recycled back to the cell surface. Alternatively, if most HLA-E is internalized with VL9 bound, it would be interesting to examine what promotes VL9 dissociation and subsequent peptide exchange. Given the highly diverse peptidome of HLA-A3, the transport kinetics of HLA-A3 molecules are also likely to be non-homogenous and more diverse than those of HLA-E. This highlights a need for future work to fully understand the difference between HLA-E trafficking patterns and those of HLA-Ia molecules with different peptide cargoes. Although we revealed the important regulatory role of HLA-E’s cytoplasmic tail in its endosomal transport, the exact mechanisms involved and possible motifs influencing transport are unclear. Furthermore, as the swapping of the cytoplasmic tail did not completely reverse the surface stability of surface HLA-I molecules and endosomal distribution, the existence of other undiscovered mechanisms, possibly differences in the recycling process, seems likely (Fig. 7). In summary, further dissection of endosomal transport pathways and the mechanisms underlying HLA trafficking and peptide association therein is required to inform the development of new approaches to facilitate the presentation of pathogen-derived peptides with HLA-E.

To conclude, this study characterized HLA-E intracellular distribution and dynamic transport process and explored the regulatory mechanism of HLA-E binding peptides and the cytoplasmic tail. The findings provide a better understanding of how HLA-E is elegantly regulated to achieve its immunological functions and give insight into HLA-E’s relative resistance to surface downregulation during infections. Importantly, the work begins to pave the way for the design of effective and safe, CMV-independent vaccines that elicit HLA-E–restricted CD8 T cell responses.

## Materials and methods

### Peptides

Peptides (VMAPRTVLL, VL9; RMYSPTSIL, RL9; and ATPLLMQAL, AL9) were purchased at a purity of 85% (GenScript), reconstituted to a final concentration of 200 mM in DMSO, aliquoted, and stored at −80°C.

### DNA constructs

All plasmids were prepared using QIAprep Spin Miniprep kits (Qiagen). Primers (Table S1) and GeneArt Strings were purchased from Life Technologies. All sequences were confirmed by Sanger sequencing using a ABI 3770. GeneArt Strings encoding HLA-E*01:03 and HLA-A*03:01 were inserted between the HindIII and BamHI sites of pEGFP-N1 (Clontech). Hybrids of HLA-E and HLA-A3 with the extracellular domains swapped (HLA-EA3 and HLA-A3E) were created by overlap extension PCR using appropriate flanking primers (EGFP-N1 F and EGFP-N1 R) with internal primers that bind to the conserved sequence that encodes the start of the HLA-E and HLA-A3 cytoplasmic tail (EA3 F and EA3 R for HLA-EA3, A3E F and A3E R for HLA-A3E) and inserted using NEBuilder HiFi DNA Assembly (New England Biolabs) following the manufacturer’s instructions.

HLA-E, HLA-EA3, HLA-A3, and HLA-A3E without the EGFP tag were amplified from the corresponding EGFP-fusion constructs using EGFP-N1 F and corresponding reverse primers (E NotI R for HLA-E and HLA-A3E, A3 NotI R for HLA-EA3 and HLA-A3).

The β2m expression plasmid was created by inserting a GeneArt string encoding β2m with a C-terminal Picornavirus 2A “slip” sequence between the HindIII and AgeI restriction sites of pEGFP-N1 so that β2m and EGFP would be expressed as separate proteins in transfected cells. Peptide expression constructs were generated by annealing complementary oligonucleotides encoding the desired peptide sequences with appropriate overhangs to allow insertion between the HindIII and AgeI sites of pEGFP-N1.

The plasmid expressing the Sec61B RUSH hook was created by inserting a GeneArt String encoding an str-Sec61B fusion into pEGFP-N1 HindIII and NotI. The HLA-SBP-EGFP RUSH reporter plasmids were created by inserting HindIII-EcoRI fragments encoding HLA-E or HLA-A3 (amplified with primers EGFP-N1 F and HLA-E EcoRI R or HLA-A3 EcoRI R, as appropriate) and an EcoRI-NotI fragment encoding an SBP-EGFP fusion (from pIRESneo3 Str-Ii+CD44-SBP-EGFP, a kind gift of Frances Brodsky, University College London, London, UK) between the HindIII and NotI sites in pEGFP-N1.

Lentiviral constructs were created by replacing the EGFP of pLenti CMV GFP Puro (a gift from Eric Campeau and Paul Kaufman; plasmid #17448; Addgene; Campeau et al., 2009) with EGFP-fusions of HLA-E, HLA-A3, HLA-EA3, and HLA-A3E amplified from the pEGFP-N1 expression plasmids with appropriate primers (pLenti E F or pLenti A3 F and pLenti R) to add the required BamHI and SalI restriction sites to their 5′ and 3′ ends.

### Cell culture and differentiation

HEK293T cells and HeLa cells were cultured in DMEM (Life Technologies), while THP-1 cells and K562 cells were cultured in RPMI 1640 Medium (Life Technologies). All culture media were supplemented with 10% heat-inactivated fetal bovine serum (Sigma-Aldrich) and penicillin/streptomycin (50 units/ml and 50 µg/ml, respectively; Life Technologies), and cells were maintained at 5% CO_2_/37°C. K562 cells stably transfected with HLA-E*01:03 (Lampen et al., 2013) were generously provided by Thorbald van Hall (Leiden University Medical Centre, Leiden, Netherlands). THP-1 cells were differentiated into macrophages by overnight incubation in the presence of 50 ng/ml PMA (Merck), followed by PMA removal with fresh media the following day.

### Transfection

HEK293T cells and HeLa cells were seeded at 50–80% confluence in 6-well plates 24 h before transfection. HEK293T cells were transfected with 1 µg of plasmid DNA using GeneJuice (Merck), and HeLa cells were transfected with 2 μg of plasmid DNA using Lipofectamine 3000 (Thermo Fisher Scientific) following the manufacturer’s instructions and were harvested after 24 h. For immunofluorescence, HeLa cells were seeded in 24-well plates and transfected with 0.5 µg of total plasmid DNA. For the RUSH assay, hook and reporter plasmids were transfected at ratios of 5:1 and 10:1 for HLA-E and HLA-A3, respectively, to ensure optimal ER trapping. When plasmids expressing peptides were included in the RUSH assay, an equal amount of the peptide minigene plasmid and the RUSH plasmids were transfected. In HEK293T cells co-transfected with HLA-E and different amounts of peptide minigene plasmid or β2m plasmid, 0.2 µg HLA-E plasmid was transfected for each sample, and the total amount of plasmid is topped up to 1 µg by the addition of EGFP plasmid.

### Construction of HeLa stable cell lines

HeLa cells stably expressing EGFP-fusions of HLA-E, HLA-A3, or the EA3 and A3E hybrids were generated by lentiviral transduction. To produce lentivirus, HEK293T cells were seeded in 6-well plates at 50% confluence 24 h before transfection. Transfection was done by cotransfecting 1 μg transfer plasmid, 0.5 μg pMD2.G envelope plasmid (a gift from Didier Trono; plasmid #12259; Addgene), and 0.5 μg pCMV-dR8.91 packaging plasmid using GeneJuice (Merck) following the manufacturer’s instructions. Media was replaced with antibiotic-free media 8 h after transfection, and viral supernatant was collected 48 h after transfection and filtered through a 0.22 µm syringe filter (Merck). HeLa cells were seeded 24 h before transduction, and the media was replaced by 0.5 ml viral supernatant and 1.5 ml antibiotic-free media containing polybrene (Santa Cruz Biotechnology) at a final concentration of 10 μg/ml. Samples were then centrifuged at 800 ×*g* for 30 min at 32°C. 24 h after transduction, the virus was removed and 2 ml fresh media was added. 72 h after transduction, media containing puromycin (Life Technologies) was added at a final concentration of 2 μg/ml for selection for a week.

### Flow cytometry

For surface staining, cells were washed twice with Dulbecco’s PBS (DPBS; Sigma-Aldrich), stained for 20 min on ice in 100 μl DPBS, washed twice in DPBS, and fixed for 20 min on ice in 100 µl Cytofix (BD Biosciences). For intracellular staining, cells were washed twice with DPBS, fixed, and permeabilized in 100 μl Cytofix/Cytoperm Solution (BD Biosciences) for 20 min on ice, washed twice in Perm/Wash buffer (BD Biosciences), stained for 20 min on ice in 100 μl Perm/Wash buffer, washed twice with Perm/Wash buffer, once with DPBS, and resuspended in DPBS. Cells were acquired using a Cyan ADP Analyser (Beckman Coulter) or an Attune NxT Flow Cytometer (Thermo Fisher Scientific), and data were analyzed using FlowJo 10.4. Details of antibodies used are listed in Table S2.

### Endo H assay

Cells were lysed by incubating for 20 min on ice in RIPA buffer (10× RIPA Lysis buffer [Merck] diluted in double-distilled H_2_O, with the addition of protease inhibitor cocktail [Roche], PhosSTOP [Roche], and PMSF [Stratech Scientific]). Lysates were centrifuged at 13,000 rpm (4°C) for 15 min to remove cell debris. Samples were then treated with 1,000 U of Endo Hf (New England Biolabs) for 2 h at 37°C following the manufacturer’s protocol. Equal volumes of lysates were mixed with LDS Loading buffer (Life Technologies), run on 4–12% Bis-Tris gels (Life Technologies) in 3-(*N*-morpholino)propanesulfonic acid buffer (Life Technologies), and then transferred to polyvinylidene difluoride membrane (Merck) in 2× NOVEX Transfer buffer (Life Technologies) on a Trans-Blot SD semi-dry transfer cell (Bio-Rad). Membranes were blocked for 1 h at room temperature in blocking buffer (5% skim milk [VMR], 0.05% Tween-20 [Sigma-Aldrich] in DPBS), then incubated for 1 h at room temperature with primary antibodies diluted in blocking buffer. For the HeLa stable cell lines, mouse anti-EGFP was used to detect HLA-E_EGFP or HLA-A3_EGFP. For THP1-derived macrophages, MEM-E/02 and HC10 were used to detect endogenous HLA-E and HLA-I, respectively. Membranes were washed three times with DPBS+0.05% Tween-20 (10 min each), incubated with IRDye 800CW secondary antibody diluted in blocking buffer for 1 h at room temperature, washed as before, and imaged using a LICOR Odyssey X. Details of the antibodies used are listed in Table S2.

### Immunofluorescence staining

Immunofluorescence staining was carried out at room temperature, with the samples protected from light. Cells were washed twice with DPBS (Sigma-Aldrich) and fixed with 4% formaldehyde (16% formaldehyde [Thermo Fisher Scientific] diluted in DPBS) for 15 min. For the RUSH assay, biotin diluted in media was added to reach a final biotin concentration of 50 μM, and cells were directly fixed with 4% formaldehyde at different time points after biotin addition without DPBS washing. After three washes with DPBS, samples were permeabilized and blocked by incubating for 1 h in blocking buffer (DPBS containing 0.5% saponin [Merck] and 2% bovine serum albumin [Merck]), stained with primary antibody diluted in blocking buffer for 1.5 h, washed quickly three times with blocking buffer, stained with secondary antibody diluted in blocking buffer for 2 h, washed as before with the blocking buffer, washed twice with DPBS, and then mounted with DAPI fluoromount-G (Cambridge Bioscience). Images were obtained using a Zeiss LSM880 inverted confocal laser scanning microscope with a Plan-Apochromat 63×/1.4 oil objective. Images were processed and analyzed in Fiji/ImageJ. ROI was selected for individual cells excluding the nuclear region, and colocalization was calculated using the Coloc2 plug-in to determine PCC. Details of the antibodies used are listed in Table S2.

### Live cell imaging

Live cell imaging was adapted from Boncompain et al. (2012) and performed at 37°C in a thermostat-controlled chamber using a Zeiss LSM880 inverted confocal laser scanning microscope with a Plan-Apochromat 63×/1.4 oil objective. Briefly, HeLa cells were seeded on μ-Slide 8-well HIGH glass bottom plate (Thistle Scientific) 24 h before transfection with Sec61B_str and HLA-E_SBP_EGFP. 24 h after transfection, biotin diluted in media was added to reach a final biotin concentration of 50 μM, and images were acquired every minute for 120 min. Videos were processed in Fiji/ImageJ.

### Acid-stripping assay

To analyze the dynamics of MHC-I transport in THP1-derived macrophages, cells were washed twice with cold DPBS and resuspended in citric acid buffer (pH 3.0) for 3 min on ice to strip off surface MHC-I proteins. Cells were then neutralized with 20 volumes of cold media and washed twice with cold DPBS. Cells were resuspended in prewarmed media and incubated at 37°C to allow for the recovery of surface MHC-I. BFA (Cambridge Bioscience) was added at 10 µg/ml to some samples to inhibit the antegrade transport of MHC-I. Cells were collected at appropriate times and fixed with 100 µl Cytofix (BD Biosciences). Surface expression of HLA-E or total MHC-I was further assessed by flow cytometry. The amount of protein recovered was calculated as the percentage of the initial surface signal before acid stripping.

To analyze the internalization rate of different HLA-I molecules, HeLa cells or THP1-derived macrophages were stained on ice for 30 min with 3D12-APC (HLA-E), GAP.A3-APC (HLA-A3), or W6/32-APC (HLA-I). Cells were washed twice with cold DPBS to remove unbound antibodies and then incubated at 37°C to allow for internalization in the presence of 0.3 mM primaquine (Enzo Life Sciences) for different time lengths. Acid stripping was performed as described above, and samples were fixed with 100 µl Cytofix (BD Biosciences) and then assessed by flow cytometry. The amount of protein internalized was calculated as the percentage of the initial surface signal before acid stripping. Details of the antibodies used are listed in Table S2.

### BFA decay assay

Cells were incubated with BFA (Cambridge Bioscience) for different time lengths at a final concentration of 10 µg/ml, with or without a 3-h preincubation of exogenous peptides (100 μM), and surface expression of HLA-E or total MHC-I protein was assessed by flow cytometry. Protein transport was stopped by keeping all samples strictly at 4°C during surface staining. Surface stability was calculated as the percentage of the initial surface signal without BFA incubation. Details of the antibodies used are listed in Table S2.

### Statistical analysis

Data were analyzed in GraphPad Prism 9 and presented as mean ± SD (ns, not significant; *, P < 0.05; **, P < 0.01; and ****, P <0.0001). Two-tailed Student’s *t* test with Welch’s correction (two groups, unpaired or paired) or one-way ANOVA with Tukey’s post-hoc test (three or more groups) was performed for comparison between groups.

### Online supplemental material

Fig. S1 characterizes the intracellular distribution of HLA-E in THP1-derived macrophages. Fig. S2 contains the validation and more figures for the RUSH system. Fig. S3 shows the functions of the high-affinity HLA-E binding peptide VL9. Fig. S4 shows EGFP tagging does not affect the functions of the HLA-E cytoplasmic tail. Fig. S5 shows how the HLA-E cytoplasmic tail affects surface turnover. Video 1 shows real-time imaging of the synchronized trafficking of HLA-E-SBP-EGFP. Table S1 lists primers for plasmid construction. Table S2 lists antibodies.

## Supplementary Material

Table S1 lists primers for plasmid construction.

Table S2 lists antibodies.

SourceData F1 contains original blots for Fig. 1.

SourceData FS1 contains original blots for Fig. S1.

## Data Availability

The data underlying all the figures are available in the published article and its online supplemental material.
