# Supplementary material for: Intracellular trafficking of HLA-E and its regulation
Source: J Exp Med. 2023 May 4;220(8):e20221941. doi: 10.1084/jem.20221941 (PMC10165540; doi:10.1084/jem.20221941)
Supplement: Table S1 — lists primers for plasmid construction. [file JEM_20221941_TableS1.docx]

Table S1. Primers for plasmid construction

| Name | Sequence (5′–3′) |
| --- | --- |
| EGFP-N1 F | CTACCGGACTCAGATCTCGAGCTCA |
| EGFP-N1 R | GGTATGGCTGATTATGATCTAGAGTCGC |
| EA3 F | GAGCTGTGGTTGCTGCTGTGATATGGAGGAGGAAGAGCTCAGATAGAAAAGG |
| EA3 R | CCTTTTCTATCTGAGCTCTTCCTCCTCCATATCACAGCAGCAACCACAGCT |
| A3E F | GGTCGCTGCCGTGATGTGGAGGAAGAAGAGCTCAGGT |
| A3E R | ACCTGAGCTCTTCTTCCTCCACATCACGGCAGCGACC |
| E NotI R | TCGCGGCCGCTCACAAGCTGTGAGACTCAGACCCCTGG |
| A3 NotI R | TCGCGGCCGCTCACACTTTACAAGCTGTGAGGGACACATCAGA |
| HLA-E EcoRI R | CGGAATTCGACAAGCTGTGAGACTCAGACCCCAGG |
| HLA-A3 EcoRI R | CGGAATTCGACACTTTACAAGCTGTGAGGGACACATCAG |
| pLenti E F | AGAAGACACCGACTCTAGAGGATCCGCCACCATGGTAGATGGAACCCTCCT |
| pLenti R | TGTAATCCAGAGGTTGATTGTCGACTCACTTGTACAGCTCGTCCATGCCG |
| pLenti A3 F | AGAAGACACCGACTCTAGAGGATCCGCCACCATGGCCGTCATGGC |
