# Supplementary material for: Intracellular trafficking of HLA-E and its regulation
Source: J Exp Med. 2023 May 4;220(8):e20221941. doi: 10.1084/jem.20221941 (PMC10165540; doi:10.1084/jem.20221941)
Supplement: Table S2 — lists antibodies. [file JEM_20221941_TableS2.docx]

Table S2. Antibody list

| Antibody | Target | Final Concentration (ng/μl) | Assay | Source |
| --- | --- | --- | --- | --- |
| APC-conjugated 3D12 | HLA-E, HLA-EA3 | 2.5 | FC | BioLegend, 342606 |
| APC-conjugated GAP.A3 | HLA-A3, HLA-A3E | 2.5 | FC | Life Technologies, 17-5754-42 |
| APC-conjugated W6/32 | HLA-I | 2.5 | FC | Life Technologies, 17-9983-42 |
| APC-conjugated BB7.2 | HLA-A2 | 2.5 | FC | BioLegend, 343308 |
| anti-EGFP | EGFP | 1 | WB | Proteintech, 66002-1 |
| MEM-E/02 | HLA-E | 1 | WB | Enzo Life Sciences,  ALX-805-701-C100 |
|  |  | 10 | IF |  |
| HC10 | HLA-I | 0.5 | WB | purified from hybridoma supernatant |
| IRDye 800CW  donkey anti-mouse | mouse IgG | 0.05 | WB | LiCor, P/N: 926-32212, |
| anti-calnexin | calnexin | 2 | IF | Abcam, ab22595 |
| anti-EEA1 | EEA1 | 2 | IF | Abcam, ab2900 |
| anti-Rab7 | Rab7 | 0.05 | IF | Abcam, ab137029 |
| anti-Rab11 | Rab11 | 1 | IF | Life Technologies, 71-5300 |
| anti-LAMP1 | LAMP1 | 1 | IF | Abcam, ab24170 |
| anti-GM130 | GM130 | 0.25 | IF | Abcam, ab52649 |
| goat anti-rabbit  Alexa Fluor 568 | rabbit IgG | 2 | IF | Abcam, ab175471 |
| donkey anti-rabbit  Alexa Fluor 647 | rabbit IgG | 2 | IF | Abcam, ab150075 |
| goat anti-mouse  Alexa Fluor 488 | mouse IgG | 2 | IF | Abcam, ab150113 |
| W6/32 | HLA-I | 2 | IF | BioLegend, 311402 |
| S8E4 | anti-streptavidin | 1 | IF | Novusbio, NB120-10023 |

^*FC, flow cytometry; WB, western blotting; IF, immunofluorescence.^
