# Supplementary figures and images for: Intracellular trafficking of HLA-E and its regulation
Source: J Exp Med. 2023 May 4;220(8):e20221941. doi: 10.1084/jem.20221941 (PMC10165540; doi:10.1084/jem.20221941)

Figure 1 – B  
Uncropped gel

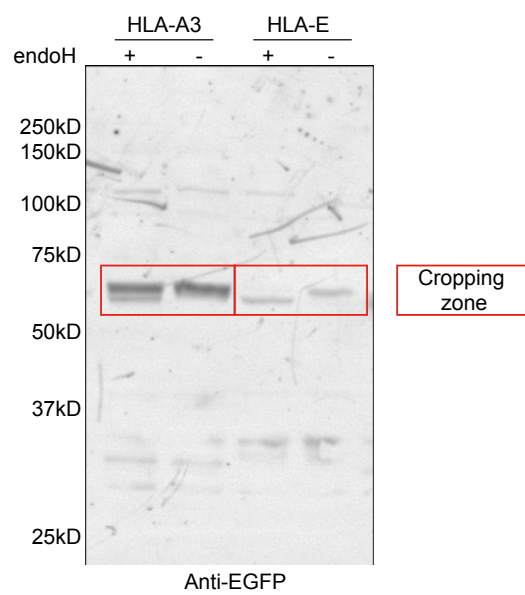

Supplement: SourceData F1 — contains original blots for Fig. 1. [file JEM_20221941_SourceDataF1.pdf]

Figure S1 – B  
Uncropped gel

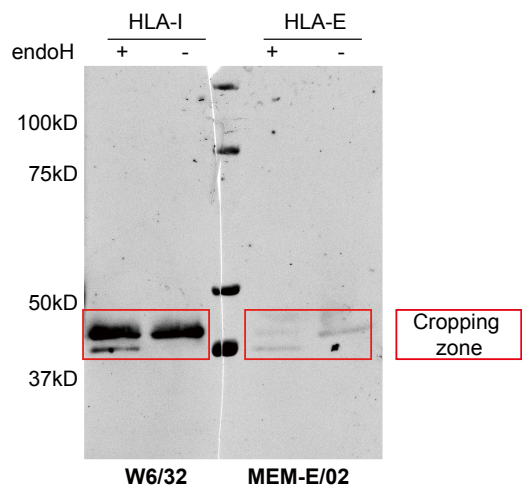

Supplement: SourceData FS1 — contains original blots for Fig. S1. [file JEM_20221941_SourceDataFS1.pdf]
